# Methods to detect selection history in a population under ongoing directional selection

**DOI:** 10.1093/genetics/iyag136

**Published:** 2026-05-27

**Authors:** Anne C M Jansen, Mario P L Calus, Yvonne C J Wientjes

**Affiliations:** Animal Breeding and Genomics, Wageningen University and Research, Wageningen 6700 AH, The Netherlands; Animal Breeding and Genomics, Wageningen University and Research, Wageningen 6700 AH, The Netherlands; Animal Breeding and Genomics, Wageningen University and Research, Wageningen 6700 AH, The Netherlands

**Keywords:** selection history, complex traits, pigs, indirect selection

## Abstract

The aim of animal breeding is to select the genetically best animals to improve the performance of future generations for a specific breeding goal. Knowledge of the (indirect) selection history of new traits is valuable before adding it to the breeding goal. Two methods have been developed to assess the selection history: BayesS estimates a parameter (*s*) that reflects the relationship between estimated additive effects and minor allele frequency of markers, while $\hat{G}$ calculates the expected genetic change of a trait based on allele frequency changes and estimated additive effects of markers. We evaluated the performance of both methods in an animal breeding context, focusing on their ability to detect selection for a trait with low heritability under direct and indirect selection. We simulated direct selection in a commercial pig breeding program under phenotypic selection, with varying heritabilities (0.05, 0.1, and 0.3) across 30 generations. In addition, indirect selection was simulated using a correlated trait with heritability 0.05 and a genetic correlation of 0.4 or 0.7 to a trait with heritability 0.1 under direct selection. Both methods were able to detect selection, where higher heritabilities and a larger sample size (for *s*-value estimation) or a longer selection interval (for $\hat{G}$) increased detectivity. The detectivity of indirect selection was limited; only $\hat{G}$ identified selection in some scenarios, where estimating marker effects in the starting generation increased detectivity. Overall, we observed that both methods have potential to identify selection but that the preferred method depended on the available data of that population.

## Introduction

The aim of animal breeding is to select the genetically best animals in the current generation to improve the performance of future generations for a specific breeding goal. However, direct selection on breeding goal traits can cause indirect selection on traits not included in the breeding goal. The traits included in the breeding goal are determined based on societal and consumers’ demands. In the past, the focus of animal breeding programs was mainly on production traits, while currently there is more focus on balanced breeding, in which the aim is to simultaneously improve productivity, efficiency, environmental impact, animal health and welfare, food quality and safety, and genetic diversity (Olesen et al. 2000; Neeteson-van Nieuwenhoven et al. 2013; Boichard et al. 2015). With this change in breeding goal, new traits have become of interest. Knowledge of the (indirect) selection history of these traits would be insightful before the trait is included in the breeding goal. Strong ongoing indirect selection on a trait would be an indication that this trait has a nonzero genetic correlation to the other breeding goal traits. Understanding the history of selection on these traits is crucial to monitor unforeseen changes caused by indirect selection pressures.

Two methods have been proposed that can assess the selection history of a trait using allele frequency data and estimated additive marker effects: BayesS and $\hat{G}\;$(Beissinger et al. 2018; Zeng et al. 2018). BayesS estimates a parameter (*s*) that reflects the relationship between estimated additive marker effects and minor allele frequency (MAF) (Zeng et al. 2018). Those estimated additive marker effects reflect the average change in the phenotype when 1 allele at a locus is replaced by the other segregating allele. If a trait is under selection, the selection pressure will be strong on alleles with a large effect, thereby increasing the frequency of favorable alleles and decreasing the frequency of unfavorable alleles (Pritchard 2001). Under positive selection, the frequency of loci with large effects is increased in the population and these are therefore more common. Accordingly, a positive value for *s* is expected for this situation. If a trait is under negative selection, the allele frequency of large-effect loci will be reduced in the population. This results in large-effect loci with low MAFs, which is reflected by a negative value for *s* (Zeng et al. 2018; Ashraf and Lawson 2021).

$\hat{G}$ uses the change in allele frequency between different generations of all loci combined with the estimated additive marker effects to evaluate the direction of selection (Beissinger et al. 2018). This method calculates the expected response to selection for all separate loci by multiplying the allele frequency change of a locus by its estimated additive effect. These values per locus are combined to get a genome-wide estimate for the response to selection of the past (Beissinger et al. 2018). It is expected that the allele frequency will change proportionally to the size and in the direction of the estimated additive marker effects (Wright 1937; Beissinger et al. 2018).

BayesS was originally described to detect selection in human populations, which undergo strictly natural selection (Zeng et al. 2018). This method, or equivalent approaches, have occasionally been applied to artificially selected populations (ie tilapia, maize, cattle, and pigs) (Joshi et al. 2021; Neshat et al. 2023; Palali Delen et al. 2023; Zhang et al. 2025). However, detailed studies on how well this method works for finite populations under strong selection are still missing. $\hat{G}$ has been applied to breeding populations in different studies, resulting in the significant detection of selection in plants (Morales et al. 2021; Tilhou et al. 2022) and pigs (Chen et al. 2022). These studies investigated selection for highly heritable traits over various selection intervals. However, we want to determine whether there has been indirect selection on novel traits through selection on the breeding goal, for which the selection accuracy is lower and would be comparable to phenotypic selection for low heritability traits. Therefore, our interest was in the performance of this method for lower heritabilities.

The aim of this study is to evaluate the performance of estimating *s*-values (based on BayesS) and $\hat{G}$ in an animal breeding context, focusing on their ability to detect direct and indirect selection for a trait with a low heritability. Moreover, the aim is to identify the required amount of information that is needed to reliably determine the presence of selection. Both $\hat{G}$ and *s*-value estimation were applied to a simulated dataset of a commercial pig breeding program under phenotypic selection, with varying heritabilities and number of generations under selection to investigate their performance. The outcomes provide insights into the strengths and limitations of both these approaches and their potential for application in animal breeding programs to identify previous selection for traits, which may have been indirect by nature, based on estimated additive effects and allele frequency data.

## Material and methods

### Simulated population structure

**Fig. 1.**
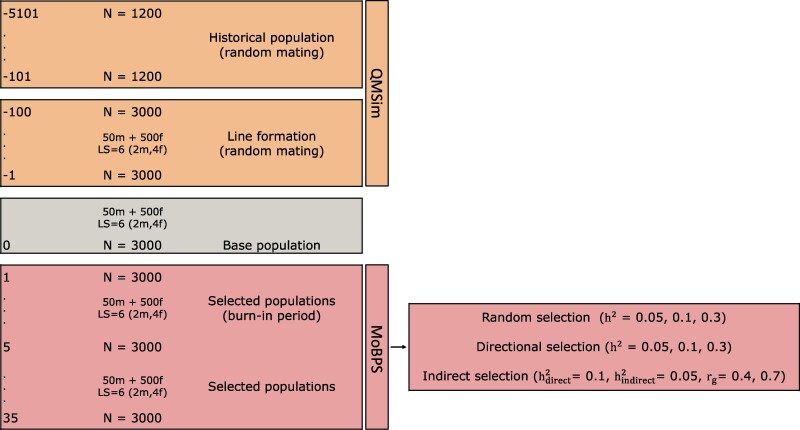
Overview of pig breeding simulation.

We simulated a commercial breeding population of pigs. First, a historical population was simulated to create genotypes for the base population using QMSim (v.2.0) (Sargolzaei and Schenkel 2009) (Fig. 1). The historical population started with 1,200 animals (600 males and 600 females). These animals were randomly selected and mated for 5,000 discrete generations to establish realistic linkage disequilibrium (LD). From the last generation of the historical population, 50 males and 500 females were randomly selected and randomly mated to create a pig line. To establish the line, random mating was continued for 100 generations while randomly selecting 50 males and 500 females each generation. The litter size was set to 6, consisting of 4 females and 2 males. These numbers represented the number of selection candidates that were assumed to be randomly chosen from the total litter (Lillehammer et al. 2011; Wientjes et al. 2020). The simulations were set up to generate an allele frequency pattern and LD comparable to that in pig breeding populations.

After line formation, the data were further processed using R (v.4.2.3) (R Core Team, 2023) and MoBPS (v.1.11.53) (Pook et al. 2020). From the last generation of line formation, 50 males and 500 males were randomly selected and mated to become parents of the base population. Thereafter, 2 different selection strategies were applied, namely directional selection based on own performance and random selection. A burn-in period of 5 generations was used to allow the population to adjust to the specific selection strategy. Thereafter, selection was continued for another 30 generations, and those generations were used in later analysis. All scenarios were replicated 50 times, and in each replicate a unique base population was created. To ensure comparability between selection scenarios, the same base population and seed were used for the different selection strategies within a replicate. This means that the same causal loci were sampled for the random and directional scenarios and for the 3 different heritability scenarios within each replicate. This was done to make sure that the observed differences between the random selection scenarios were strictly because of the difference in heritability and not due to sampling differences, while the differences between the directional selection scenarios were because of the differences in heritability and selection.

### Genome size

**Fig. 2.**
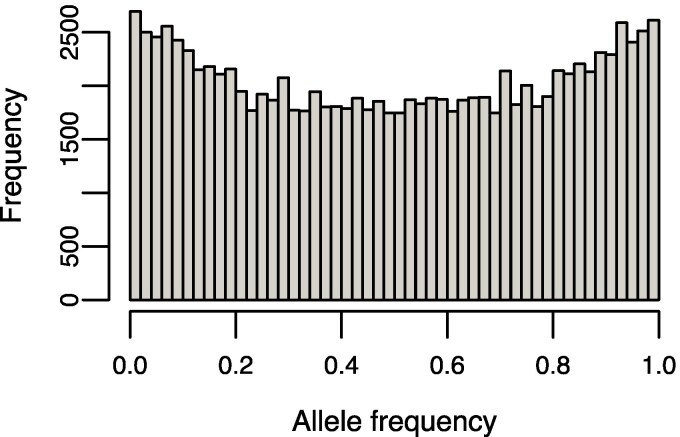
Allele frequency distribution in one replicate in the last generation of line formation.

**Fig. 3.**
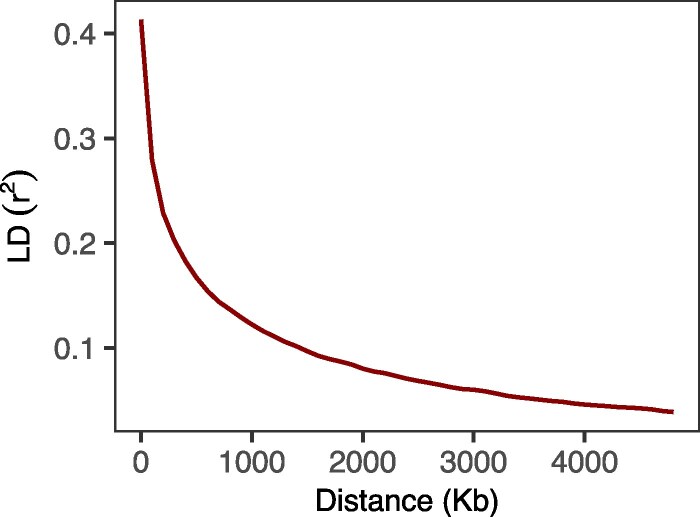
Linkage disequilibrium decay of one replicate in the last generation of line formation.

Just like the pig genome, 18 chromosomes were simulated, and the map length of each chromosome was set to be similar to that of pigs (Tortereau et al. 2012). Each chromosome was assigned 20,000 loci in the first generation of the historical population, which were randomly spaced and had a randomly sampled allele frequency. The recurrent mutation rate was 2.5 × 10^−5^ in the historical population. Random assortment of chromosomes represented variation due to Mendelian sampling. From the last generation of the historical population, loci with a MAF higher than 0.01 were selected and no new mutations were introduced after this point. After line formation, the allele frequency distribution of segregating loci in the base population was slightly U-shaped (Fig. 2), which was caused by genetic drift and is in line with the expectations for random mating populations (Walsh and Lynch 2018), and was also seen for whole-genome sequence data in livestock (Brøndum et al. 2014; Daetwyler et al. 2014; Heidaritabar et al. 2016). The LD decay in the base population (Fig. 3) was comparable to the LD in commercial pig breeding populations (Badke et al. 2012; Veroneze et al. 2013).

From the segregating loci in the base population, 2,000 loci were randomly sampled to become causal loci. From the remaining segregating loci over all chromosomes, 20,000 markers were selected in total by first dividing all loci into 100 bins based on their allele frequency, where bin 1 contained alleles with a frequency between 0 and 0.01, bin 2 between 0.01 and 0.02 and so on. From each bin, 200 loci were randomly sampled as markers, leading to a uniform allele frequency distribution for markers. This was representative of SNP arrays used in livestock (Matukumalli et al. 2009; Kranis et al. 2013).

### Phenotypes

For directional and random selection, the additive effects for all causal loci were sampled from a gamma distribution with shape parameter 0.4. Those effects were randomly assigned a positive or negative sign, using the criteria that there were always 1,000 positive and 1,000 negative effects. The additive effects were multiplied by the allele counts of the causal loci (0, 1, or 2), to get the additive genetic values of each individual. The additive effects of these loci were scaled in MoBPS to ensure a mean of 0 and an additive genetic variance in the base population equal to the target heritability. Consequently, the phenotypic variance was standardized to 1, and the environmental variance in the base population was defined as $1-h^{2}$. This standardization of the additive genetic variance ensured that the causal effects were on a comparable scale across replicates.

For the indirect selection scenarios, a trait was simulated that was genetically correlated to the trait under direct selection with a heritability of 0.1. To simulate the genetic correlation between traits, we assumed that the same causal loci were affecting both traits, ie assuming pleiotropy. This was achieved by scaling the simulated additive effects of the direct trait to a normal distribution with mean 0 and standard deviation of 1 and multiplying these effects with the Cholesky decomposition of the covariance matrix containing the genetic variance of both traits (set to be 1) on the diagonal and the genetic correlation between traits (set to 0.4 or 0.7) on the off-diagonal. The heritability was set to 0.05 in the base population, and environmental deviations for the correlated trait were again sampled from a normal distribution with mean 0 and variance $\sigma_{E}^{2}$. The phenotypes were simulated by adding the environmental deviations to the additive genetic values.

### Selection

Two forms of selection, ie random selection and directional selection were simulated for 30 generations after the 5-generation burn-in period. Moreover, 3 heritabilities (0.05, 0.1, and 0.3) were simulated. This resulted in 2 × 3 = 6 scenarios, with 50 replicates per scenario (Table 1). Indirect selection was simulated by creating a trait that correlated to the directly selected trait with heritability 0.1. The correlated trait had a heritability of 0.05, and a genetic correlation of 0.4 or 0.7 to the trait under direct selection.

**Table 1.** Overview of the selection strategies.

Selection type	*h* ^2^	No. of gen	Replicates	Selection	*h* ^2^ correlated trait	Genetic correlation
Directional selection	0.3	30	50	Phenotypes		
Directional selection	0.1	30	50	Phenotypes	0.05	0.4, 0.7
Directional selection	0.05	30	50	Phenotypes		
Random selection	0.3	30	50	Random		
Random selection	0.1	30	50	Random		
Random selection	0.05	30	50	Random		

### $\hat{G}$ method for selection

The first method that we tested was $\hat{G}$, developed to detect the selection history of traits based on the expected genetic gain, resulting from the change in allele frequency of markers and their estimated additive effect (Beissinger et al. 2018; Mahmoud et al. 2023). $\hat{G}$ was calculated as $\hat{G}=\sum_{\;j=1}^{m}\;\Delta_{j}\alpha_{j}$, where $\Delta_{j}$ is the change in allele frequency at locus *j*, $\alpha_{j}$ is the estimated effect of this locus *j*, and *m* is the number of genotyped markers. The method used the change in allele frequency between 2 time points or generations. In our simulation, we applied it to varying numbers of generations to detect selection over 1, 5, 10, 15, and 20 generations, looking back from generation 30 (ie to detect selection over 20 generations, the change in allele frequency was determined between generation 10 and 30). The marker effects of segregating loci (ie *α*) of the SNP array were estimated in generation 30 using rrBLUP in MoBPS.

To assess if $\hat{G}$ was significantly different from zero, the value was compared with a permuted null distribution, for which 1,000 permutations were used (Beissinger et al. 2018). The Ghat R-package was used to determine the number of independent markers based on LD in the starting generation, with a maximum window of 500 kb and a maximum LD-cutoff threshold, *r*^2^, of 0.03, as is recommended for this data structure (Mahmoud et al. 2023). This value was used to adjust the normal null distribution that resulted from the permutations as: $N\left(\bar{{\hat{G}}_{\mathrm{perm}}},\;SE({\hat{G}}_{\mathrm{perm}})\sqrt{\frac{m}{m_{\mathrm{ind}}}}\right)$, where $\bar{{\hat{G}}_{\mathrm{perm}}}$ is the mean of the $\hat{G}$ values that were estimated for each permutation, *m* is the number of markers, and *m*_ind_ is the number of independent markers. Thereafter, the observed $\hat{G}$ was compared with this null distribution to get a *P*-value. The resulting *P*-value was considered significant when <0.05. A $\hat{G}\;$ value that was significantly greater than the permuted mean indicated positive directional selection, and a value significantly lower than the permuted mean indicated negative directional selection. When $\hat{G}$ was not significantly different from the permuted mean, there was no significant evidence for selection.

### The *s-*value estimation

The second method aims to estimate a parameter *s* that reflects the relationship between the estimated additive effects of loci and their MAF at one time point in a population (Zeng et al. 2018). This relationship provides information about ongoing selection in a population. In case, the MAF and estimated additive effects are unrelated, the *s-*value would be zero. A value for parameter *s* <0 reflects that the effects of rare loci are, on average, larger than the effects of common loci, which is seen when large-effect loci are kept at low frequencies by negative selection. Contrarily, when *s* >0, the common loci have larger effects than rare loci and this is expected in human populations when selection increases the frequency of large-effect loci by positive selection (Zeng et al. 2018; Ashraf and Lawson 2021). However, artificial selection in finite populations results in a rapid increase of beneficial alleles. The beneficial alleles with large effects rapidly increase toward fixation and consequently only remain at intermediate frequencies for a short time. This results in loci with a large effect having a relatively low MAF. Alleles with smaller effects experience a lower selection pressure and remain at higher MAF. This results in a negative relationship between the MAF and effect. Therefore, a negative value for *s* is expected for livestock populations under positive selection as well, whereas an *s*-value of zero would indicate that no selection has occurred.

A genomic restricted maximum likelihood (GREML) model was used to determine the value of *s* that best fitted the data. The following mixed model was used for the GREML analysis:


y=1μ+Zu+e

where y is a vector of phenotypes, 1 is a vector of ones, *μ* is the overall mean, Z is a design matrix linking the phenotypes to the breeding values of the animals, u is a vector of breeding values with $u\;∼\;N(0,G_{s}\sigma_{a}^{2}$) where $G_{s}$ is the genomic relationship matrix constructed using parameter *s*, and $\sigma_{a}^{2}$ the total additive genetic variance. The residuals are represented by **e**, with $e\;∼\;N(0,I\sigma_{e}^{2})$, where **I** is the identity matrix and $\sigma_{e}^{2}$ is the residual variance. The **G** matrix with the parameter *s* was calculated as:


$$G_{s}=\frac{X_{S}X_{S}^{\prime }}{\sum_{\;j=1}^{m}\;{\left[2p_{j}(1-p_{j})\right]}^{1+s}}$$

Where $X_{s}$ is a scaled genotype matrix, *m* is the number of loci, $p_{j}$ is the allele frequency of locus *j*, and parameter *s*  ∈[−1,1].

The element for individual *i* and locus *j* in the scaled genotype matrix $X_{s}$ was defined as:


$$X_{S_{ij}}=(M_{ij}-2p_{j})\cdot [2p_{j}(1-p_{j}){]}^{s/2}$$

Where **M** is the allele count matrix with dimensions *n* × *m*, where *n* is the number of individuals and *m* is the number of loci, and $M_{ij}$ is the allele count (0, 1, 2) for individual *i* at locus *j*. The notation is based on (Speed et al. 2017; Zeng et al. 2018).

We varied the values for *s* from −1 to 1 with increments of 0.1, ie 21 values were tested for *s*. This resulted in 21 $G_{s}$**-**matrices that were differently scaled. It is good to note that with *s* = 0, the resulting $G_{s}$ matrix was equivalent to the **G** matrix obtained with method 1 described in (VanRaden 2008), and *s* = −1 resulted in the **G** matrix obtained with method 2 described in (VanRaden 2008). The $G_{s}$ matrices were calculated using calc_grm (Calus and Vandenplas 2016). The different $G_{s}$ matrices were then used in a GREML analysis using MTG2 (Lee and van der Werf 2016), resulting in a log likelihood value for each considered *s-*value (Bouwman et al. 2017; Speed et al. 2017). The *s-*value that resulted in the highest log likelihood was considered to best fit the data. Using AIC scores, it was determined whether the model fit for those identified *s-*values substantially differed from the fit for the other *s-*values. The AIC score was calculated as AIC=−2ln(L)+2k, where *k* is the number of estimated parameters and *L* is the likelihood estimate (Burnham and Anderson 2003). Then, we estimated for each tested *s-*value $\Delta \mathrm{AI}C_{s}=\mathrm{AI}C_{s}\;-\mathrm{AI}C_{\min}$, where $\mathrm{AI}C_{\min}\;$ is the model with the lowest AIC value (which is the best fitting model) and $\mathrm{AI}C_{s}$ are the AIC scores for each value for *s*. These values were evaluated based on the criteria from Burnham and Anderson (2003), where a $\Delta \mathrm{AI}C_{s}$ of 0–2 reflects that $\mathrm{AI}C_{\min}$ is not a substantially better fit than $\mathrm{AI}C_{s}$, 4–7 reflects that $\mathrm{AI}C_{\min}$ is a considerably better fit than $\mathrm{AI}C_{s}$, and >10 means that there is essentially no support for the $\mathrm{AI}C_{s}$ value. Here, we therefore considered that $\Delta \mathrm{AI}C_{s}$ scores of >10 indicated that there was a substantially better fit for $\mathrm{AI}C_{\min}$. We identified across replicates how often the optimal *s*-value differed from zero with a $\Delta \mathrm{AI}C_{s}$ score >10 and used this as the indication of selection.

### Development of selection signals

The development of the selection signal over the selected generations was further investigated to get insight into the number of generations of selection that are required before selection can be detected. Therefore, *s-*values were estimated in generations 1, 5, 10, 15, 25, and 30 (after the burn-in period of 5 generations). For each generation, the $\Delta \mathrm{AI}C_{s}$ were calculated to assess the certainty of the *s-*value estimates, providing insight into how clearly the selection signal could be identified over time.

### Ascertainment bias of SNP sampling

In the simulations of the SNP arrays, loci were sampled in allele frequency bins, resulting in a uniform allele frequency distribution on the array. In contrast, the causal loci were randomly sampled from all segregating loci, which had a U-shaped allele frequency distribution. The sampling of the SNP array could therefore have resulted in ascertainment bias, where the SNP array is not a good representation of the allele frequency distribution of the causal loci (Geibel et al. 2021). To investigate the impact of this on the estimation of the *s*-values, simulations with a heritability of 0.3 were repeated for both random and directional selection, where the SNPs for the array were selected completely at random, retaining the U-shaped allele frequency distribution. The *s-*values for these simulations were compared with the *s-*values for the simulations with a uniform SNP array. Furthermore, the *s-*values based on the 2,000 actual causal loci were determined and included in the comparisons.

### Development of MAF of causal loci by selection

To evaluate how the genetic architecture evolved under selection, we evaluated the correlation between MAF and additive effect of causal loci between generations 1 and 30 of selection. This correlation was expected to arise because of selection, as before selection this was zero. The regression coefficients of the absolute additive effect on the MAF were calculated in generation 1, 5, 10, 15, 20, 25, and 30 after the burn-in period. The mean slope was calculated over the 50 replicates for heritabilities of 0.05, 0.1, and 0.3 for directional selection.

## Results

### Evidence of selection

**Fig. 4.**
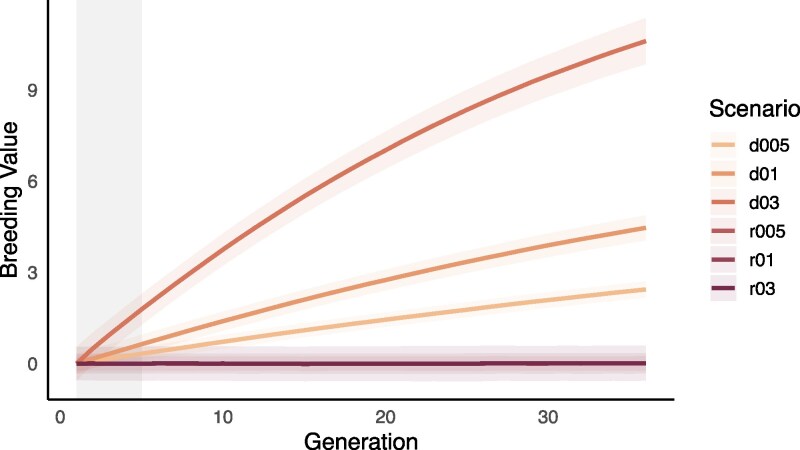
Trait development over generations for all selection scenarios: directional (d) and random (r) selection for heritabilities 0.05, 0.1, and 0.3. The lines depict the mean breeding value over 50 replicates, and ribbons indicate ±1 standard deviation across replicates. The burn-in period (first 5 generations) is shown by the shaded vertical bar.

To investigate the capability of the methods to identify selection in a pig breeding program, 30 generations of selection were simulated, preceded by 5 generations of selection as a burn-in period (Fig. 4). The average breeding values after a total of 35 generations directional selection were 2.44, 4.46, and 10.61 for heritabilities of 0.05, 0.1, and 0.3, respectively. For random selection, the average breeding values in the last generation were 0.006, 0.008, and 0.014 for heritabilities of 0.05, 0.1, and 0.3, respectively. Accordingly, random selection resulted in breeding values that showed little deviation over generations and the mean change in breeding value after 35 generations was small and not significantly different from zero (one-sample *t*-test across replicates; *P* = 0.67). Although the same animals, causal loci and additive effects were sampled across heritabilities, breeding values differed in magnitude due to scaling but followed a similar overall pattern for random selection. These results indicated that the phenotype was stable under random selection, where the observed fluctuations were only induced by genetic drift.

### $\hat{G}$ method for selection

**Fig. 5.**
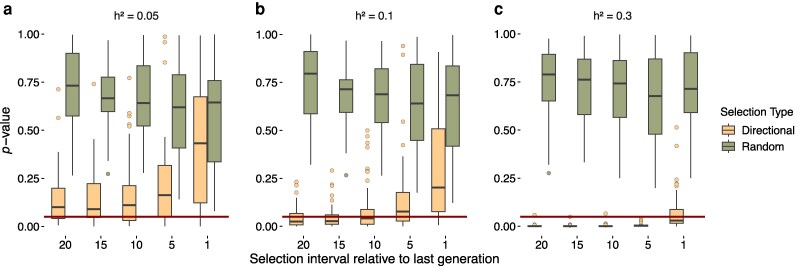
Results for $\hat{G}$ obtained for different selection types (directional and random selection), different heritabilities (0.05, 0.1, and 0.3) and different selection intervals relative to the last generation (20, 15, 10, 5, and 1).

The first method that was tested to detect selection history was $\hat{G}$, which was applied to 5 selection intervals, ie the number of generations between the 2 generations used for calculating the change in allele frequency (20, 15, 10, 5, and 1 generations), while generation 30 was always the last generation. $\hat{G}$ never detected a significant signal for selection for the random selection scenarios, as was expected (Table 2). For the trait under directional selection, $\hat{G}\;$ was able to detect a significant signal of selection in many cases, and a higher heritability resulted in a higher probability of significantly detecting selection (Fig. 5). All significant $\hat{G}$ values were positive, as was expected for the directional selection that was applied. Increasing the selection interval resulted overall in correctly identifying the selection more often. For a selection interval of 10 (ie gen. 20–30), selection was detected correctly for 98% of the replicates for *h*^2^ = 0.3, for *h*^2^ = 0.1 this was 56%, and for *h*^2^ = 0.05 this was 30%. However, increasing the selection interval further (ie for gen. 15–30 and 10–30) did not result in a strong increase in the indication of selection.

**Table 2.** Overview of number of replicates (out of 50) with a *P*-value <0.05 for $\hat{G}$ for directional and random selection with heritabilities 0.05, 0.1, and 0.3.

	Directional selection			Random selection		
Selection interval	*h* ^2^ = 0.05	*h* ^2^ = 0.1	*h* ^2^ = 0.3	*h* ^2^ = 0.05	*h* ^2^ = 0.1	*h* ^2^ = 0.3
gen. 10–30	16	34	49	0	0	0
gen. 15–30	14	35	50	0	0	0
gen. 20–30	15	28	49	0	0	0
gen. 25–30	13	19	50	0	0	0
gen. 29–30	5	10	31	0	0	0

**Fig. 6.**
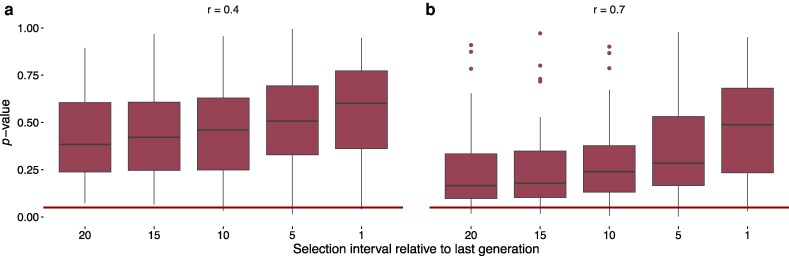
Results for $\hat{G}$ obtained for indirect selection for a trait with a heritability of 0.05, with correlation 0.4 and 0.7, resulting from direct selection on a trait with heritability of 0.1. Results are presented for different selection intervals relative to the last generation (20, 15, 10, 5, and 1).

The detectivity of $\hat{G}$ for the trait under indirect selection was generally very low (Table 3). When the genetic correlation (*r_g_*) between the indirect trait (*h*^2^ = 0.05) and the direct trait (*h*^2^ = 0.1) was 0.4, selection was detected in at most 4% of the replicates. Even though the detectivity increased when the genetic correlation was 0.7, selection was still detected in no more than 8% of the replicates (Fig. 6). Out of all significant $\hat{G}$ values, there was 1 negative $\hat{G}$ value for *r_g_* = 0.4 and a selection interval of 1 generation (*P* = 0.043). All other significant $\hat{G}$ values were positive, as was expected for directional selection.

**Table 3.** Overview of number of replicates (out of 50) with a *P*-value <0.05 for $\hat{G}$ for indirect selection on a trait with a heritability of 0.05, resulting from direct selection on a trait with a heritability of 0.1.

	Indirect selection	
Selection interval	*r*_g_ = 0.4	*r*_g_ = 0.7
gen. 10–30	0	4
gen. 15–30	0	3
gen. 20–30	1	2
gen. 25–30	1	2
gen. 29–30	2	3

### The *s-*value estimation

**Fig. 7.**
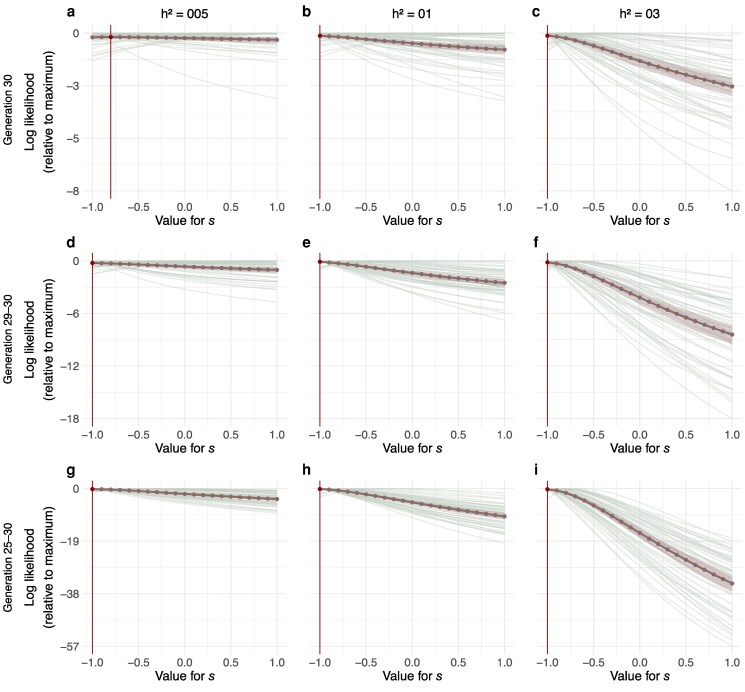
Difference in log likelihood values of different *s*-values compared with the log likelihood value of the *s*-value with the lowest log likelihood of that replicate, with *s*-values ranging between −1 and 1 for directional selection. The thin lines represent the 50 replicates, where the thick lines are the average of these replicates. The *s-*values that, on average, have the best likelihood values are highlighted with vertical lines.

The second method estimated *s-*values as an indication of selection, which were estimated for datasets containing generations 30, 29–30, and 25–30. Taking the average over all 50 replicates, the optimal *s-*value was on average between −0.8 and −1 for the directional selection scenarios (Fig. 7). Overall, an *s-*value of −1 had the highest likelihood for directional selection, except for the scenario with a heritability of 0.05 and using data of generation 30 only, where the optimal *s-*value was −0.8 on average.

The $\Delta \mathrm{AI}C_{s}$ were calculated for each replicate and *s*-value as the difference to the best fitting *s*-value, where $\mathrm{AI}C_{\min}$ was the AIC score of the *s-*value with the highest likelihood for each replicate. A $\Delta \mathrm{AI}C_{s}$ score at zero of 10 or higher indicated that the *s*-value differed substantially from an *s*-value of zero. The $\Delta \mathrm{AI}C_{s}\;$ scores revealed that for the *s-*values that were determined based on data from generation 30 only, there were no replicates where the *s*-value differed from zero, for any of the tested heritabilities (Supplementary Fig. 1; Table 4). This indicated that there was no significant evidence that the trait has been under selection. Enlarging the dataset by including the last 2 generations in the *s*-value estimation for a heritability of 0.3, resulted in 19 out of 50 *s*-values being substantially different from zero. Further enlarging the dataset resulted in $\Delta \mathrm{AI}C_{s}$ scores >10 for all heritabilities, and $\Delta \mathrm{AI}C_{s}$ scores >10 were reached for 2, 23, and 50 replicates for a heritability of 0.05, 0.1, and 0.3, respectively. With increasing heritabilities and increasing number of generations of data, the differences between log likelihood values of the best fitting *s-*value and the other *s-*values increased, thereby increasing the evidence that an *s*-value of zero was not the best fitting model.

**Table 4.** Overview of the number of replicates (out of 50) with $\Delta \mathrm{AI}C_{s}>10$, where the AIC score of the *s*-value with the highest likelihood (AIC_min_) of each replicate is compared with the AIC score for an *s*-value of zero.

	Directional selection			Random selection		
Generation	*h* ^2^ = 0.05	*h* ^2^ = 0.1	*h* ^2^ = 0.3	*h* ^2^ = 0.05	*h* ^2^ = 0.1	*h* ^2^ = 0.3
gen. 30	0	0	0	0	0	0
gen. 29–30	0	0	19	0	0	0
gen. 25–30	2	23	50	0	1	1

**Fig. 8.**
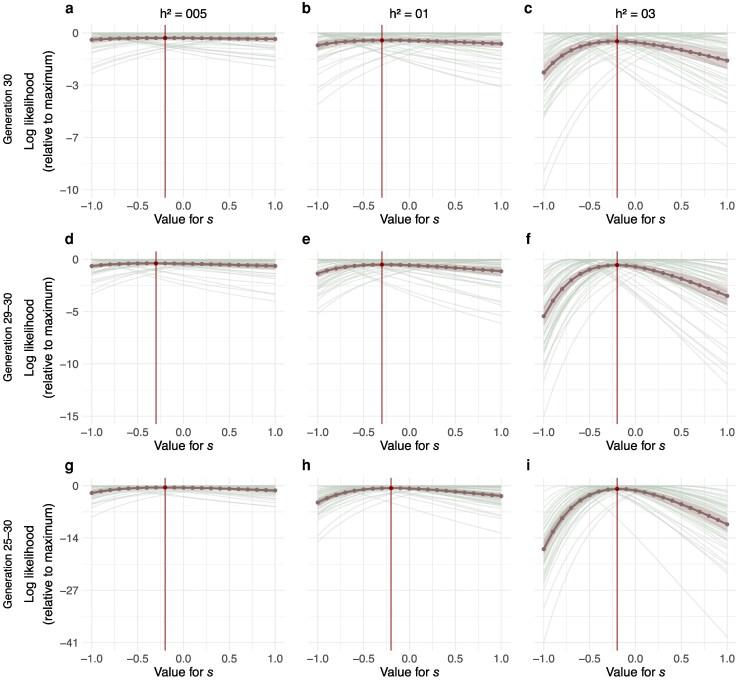
Difference in log likelihood values of different *s*-values compared with the log likelihood value of the *s*-value with the lowest log likelihood of that replicate, with *s*-values ranging between −1 and 1 for random selection. The thin lines represent the 50 replicates, where the thick lines are the average of these replicates. The *s-*values that, on average, have the best likelihood values are highlighted with vertical lines.

For random selection, the optimal *s-*value was on average across all 50 replicates between −0.2 and −0.3 (Fig. 8). The difference in the log likelihood values between the optimal *s-*value and the other *s-*values increased for higher heritabilities and an increasing number of generations of data provided, as was also seen under directional selection. The $\Delta \mathrm{AI}C_{s}$ scores revealed there were some replicates for the analysis of generation 30 only, where the best fitting *s-*value reached values >10 compared with other *s*-values (Supplementary Fig. 2). Looking at 2 and 5 generations of data, the number of replicates that reached an $\Delta \mathrm{AI}C_{s}$ score >10 compared with other *s-*values increased. However, when comparing the optimal *s-*value to an *s*-value of zero, $\Delta \mathrm{AI}C_{s}>10$ was only reached twice: once for a heritability of 0.1 and once for 0.3, with the use of the last 5 generations of data (Table 4).

**Fig. 9.**
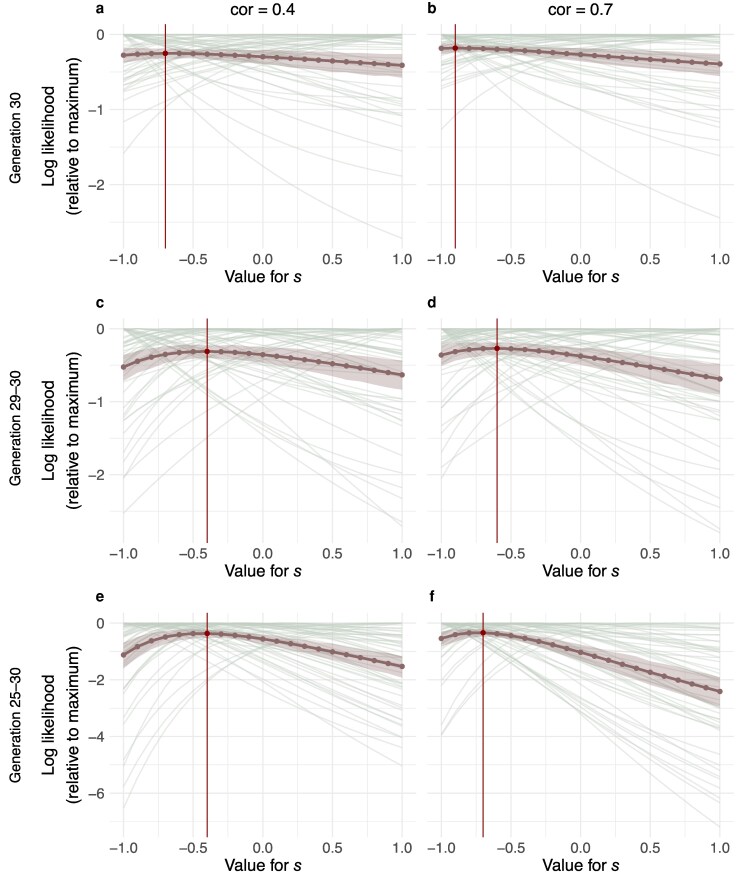
Difference in log likelihood values of different *s*-values compared with the log likelihood value of the *s*-value with the lowest log likelihood of that replicate, with *s*-values ranging between −1 and 1 for indirect selection. The thin lines represent the 50 replicates, where the thick lines are the average of these replicates. The *s-*values that, on average, have the best likelihood values are highlighted with vertical lines.

For indirect selection, the optimal *s-*value was estimated between −0.4 and −0.7 for a correlation of 0.4, and between −0.6 and −0.9 for a correlation of 0.7 (Fig. 9). The average optimum *s*-value was negative when only using data of generation 30; however, the log likelihood pattern was very flat and, in some replicates, the optimum *s-*value was positive. When more generations were included in the analysis, the number of optimal *s*-values that were positive decreased (Supplementary Fig. 3). The log likelihood pattern became less flat with an increasing number of generations of data, showing more clearly that the negative *s*-values were a better fit. However, the $\Delta \mathrm{AI}C_{s}$ scores revealed that there were no replicates where the best fitting *s-*value reached values >10 compared with other *s*-values, for any of scenarios. The *s*-value of zero could therefore not be proven to be a substantially worse fit for indirect selection in these scenarios.

### Development of selection signals

**Fig. 10.**
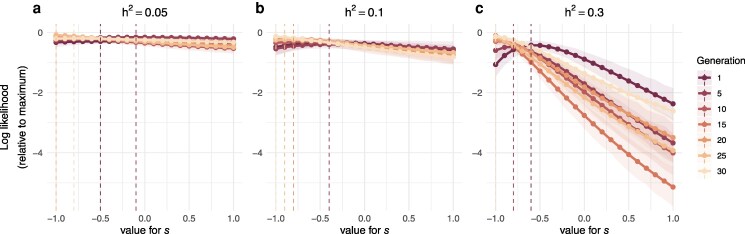
Difference in log likelihood values of different *s-*values compared with the log likelihood value of the *s*-value with the lowest log likelihood of that replicate, with *s*-values ranging between −1 to 1 for directional selection, for generations 1, 5, 10, 15, 20, 25, and 30. The lines represent the average log-likelihood values for each population. The *s-*values that, on average, have the best likelihood values are highlighted with vertical lines.

The *s-*values were estimated in generation 1, 5, 10, 15, 25, and 30 for directional selection to investigate the development of the signal to detect selection. For heritability 0.3, the estimated *s-*values became more negative for an increasing number of generations of selection. In generation 1 (the first generation after the 5 generation burn-in period), the optimal *s-*value was on average determined to be −0.6, whereas in generation 10 and onwards this was estimated to be −1 (Fig. 10). The $\Delta \mathrm{AI}C_{s}$ scores reached values >10 with at least one other *s*-value in any generation, where in most cases this significance was reached when the optimal *s-*value was (close to) −1. For this heritability of 0.3, the highest $\Delta \mathrm{AI}C_{s}$ scores and therefore the strongest selection signal, was found between generations 10–20. After this generation, the signal became weaker. Similar to heritability 0.3, the (on average) optimal *s-*value became more negative over time for a heritability of 0.1. Here, the optimal *s-*value was estimated at −0.4 in generation 1 and reached −1 in generation 30. For heritability 0.05, the optimal *s-*values did not develop uniformly in one direction as was seen for the other heritability values, and overall, the optimal *s-*values ranged between −0.1 and −1. For heritabilities of 0.05 and 0.1 the $\Delta \mathrm{AI}C_{s}$ scores only rarely reached values >10 with at least one other *s*-value and therefore the differences in model fit between the *s-*values were not substantial (Supplementary Fig. 4).

### Ascertainment bias of SNP sampling

**Fig. 11.**
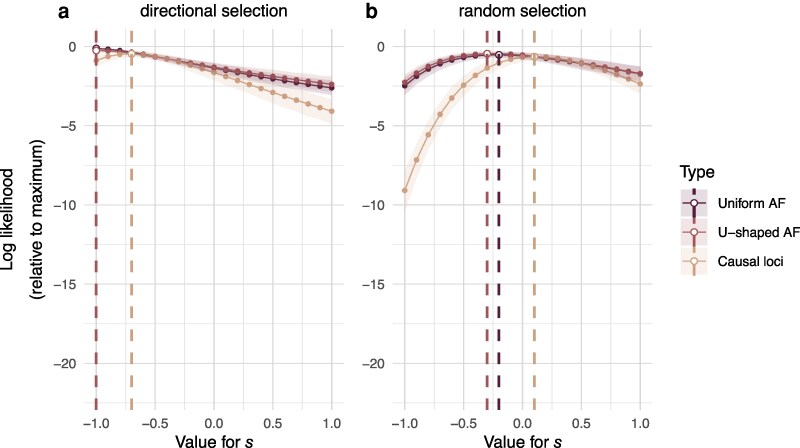
Difference in log likelihood values of different *s-*values compared with the log likelihood value of the *s*-value with the lowest log likelihood of that replicate, with *s*-values ranging from −1 to 1 for both directional and random selection, with a heritability of 0.3, after 30 generations of selection. The lines represent the average log-likelihood values for a population with a different array; u-shaped allele frequency SNP array, uniform allele frequency SNP array, only the causal loci. The *s-*values that, on average, have the optimal likelihood values are highlighted with vertical lines.

To determine if the design of the SNP array had an influence on the estimated *s-*values, the optimal *s-*values were also determined for a SNP array with U-shaped allele frequency distribution and compared with the original array with a uniform allele frequency distribution. Moreover, the *s-*values were determined directly for the causal loci. Under directional selection, the optimal *s-*value for the U-shaped as well as the uniform allele frequency SNP arrays was estimated to be −1 (Fig. 11). Yet, for the causal loci the optimal *s-*value was estimated at −0.7. For random selection, the *s-*values for the U-shaped and uniform SNP arrays were estimated at −0.3 and −0.2, respectively. Where for the causal loci in this case, it was estimated to be 0.1. This slight deviation from 0 for the causal loci could be attributed to be caused by genetic drift.

### Development of MAF of causal loci by selection

**Fig. 12.**
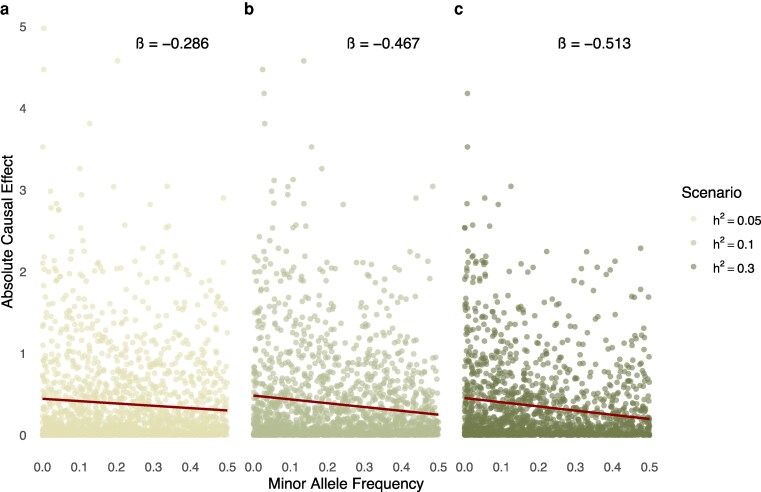
The additive effect of the causal loci vs their MAF with the regression slope for directional selection for all 3 heritabilities for 1 replicate in generation 30.

To evaluate the actual relationship between MAF of the causal loci and the additive effect under selection, we examined the regression coefficients for the MAF of causal loci with their additive effect in generations 1, 5, 10, 15, 20, 25, and 30 of directional selection. The regression coefficients became more negative over time for each heritability (Supplementary Tables 1–4). After 30 generations of selection, the mean regression coefficients were −0.297 (SD = 0.090, adj-*R*^2^ = 0.005, mean *P* = 0.025), −0.430 (SD = 0.099, adj-*R*^2^ = 0.012, mean *P* = 0.003), and −0.512 (SD = 0.083, adj-*R*^2^ = 0.020, mean *P* < 0.001) for heritabilities 0.05, 0.1, and 0.3, respectively. The regression slopes for directional selection of one replicate are depicted in Fig. 12. Although the slope became more negative with increasing heritability, the adjusted *R*^2^ values were consistently low, indicating that MAF explained only a small proportion of the variation in effects. For the random selection scenarios, for which the same causal loci were sampled, there was one common slope for the 3 heritabilities, where the mean slope was 0.0244.

## Discussion

The aim of this study was to evaluate the performance of $\hat{G}$ and estimated *s-*values to investigate the trait selection history in livestock populations. The methods were applied to a simulated pig breeding program based on either random or directional phenotypic selection for traits with heritabilities of 0.05, 0.1, and 0.3. Furthermore, an indirectly selected trait (*h*^2^ = 0.05), which was correlated to a trait under directional selection (*h*^2^ = 0.1), was simulated with a correlation of 0.4 or 0.7. Results showed that $\hat{G}$ was able to accurately detect direct selection for a trait with a heritability of 0.3 and a selection interval of at least 5 generations. Although the detectivity decreased for lower heritabilities, $\hat{G}$ did not detect a significant signal for random selection. For indirect selection, $\hat{G}$ rarely detected selection. For the estimated *s*-values, a value of zero reflected no selection, and a deviation from zero with $\Delta \mathrm{AI}C_{s}>10$ was considered a significant indication of selection. Under directional selection, optimal *s-*values were generally estimated close to −1, with higher ΔAIC scores and more frequent strong deviations from zero for higher heritabilities, and when more generations of data were included. For random selection, optimal *s*-values were slightly below zero and only in some rare cases the optimal *s*-value a considerably better fit to the data than an *s*-value of zero. The optimal *s*-values under indirect selection did not deviate from an *s*-value of zero, even though the inclusion of more data were beneficial.

### Relating the simulations to real breeding populations

In our simulation, we applied 30 generations of selection to represent the long-term selection that has been applied in commercial pig breeding programs. The nucleus populations of breeding companies have undergone continuous selection for meat production and reproductive traits for several decades, corresponding to around 30 discrete generations of selection (Amills et al. 2010; Merks et al. 2012). The selection history simulated here is therefore broadly comparable to that of real pig breeding populations.

This study has focused on the detectivity of selection in traits with low to moderate heritabilities (0.05, 0.1, and 0.3). Traits with heritabilities in the range of 0.05 to 0.1 in pigs represent common reproductive traits (ie total number born, number born alive, number born dead, number of stillborn piglets, number of mummies, litter weight, and piglet birth weight) (Schneider et al. 2012; Ye et al. 2018; Sell-Kubiak 2021; 2022; Ogawa et al. 2022), while traits with a heritability of 0.3 represent production traits in pigs (ie daily gain, feed intake, feed efficiency, back fat thickness, and muscle depth) (Bergsma et al. 2013; Godinho et al. 2018; Ogawa et al. 2022). Insight into the selection history of traits known to be under selection can be a valuable addition to current practices, where the impact of selection is generally only assessed based on the genetic trend, which shows how the average estimated breeding values change across generations. Selection history can therefore aid in the evaluation of the applied selection index and whether or not selection weights should be adjusted.

It is good to note that we focused on traits with a low heritability for the indirect selection scenarios. Furthermore, we only applied phenotypic selection. Genomic selection could increase the accuracy of selection, resulting in higher detectivity of selection for both the direct and indirect trait. Therefore, the detectivity in real breeding populations could be better than in our simulations show.

### Relation between MAF and additive effects under selection

In populations under natural selection, negative (purifying) selection acts against mutations with large deleterious effects and prevents them from becoming frequent in a population (Pritchard 2001; Eyre-Walker 2010). These deleterious mutations are kept at low frequency in the population by the principles of mutation-selection balance, resulting in the negative relationship between MAF and additive effects, as is often observed in human populations (Speed et al. 2017; Timpson et al. 2018; Zeng et al. 2018; Speed et al. 2020; Zeng et al. 2021). In contrast, directional (positive) selection acts to increase beneficial alleles in the population, potentially leading them to fixation (Walsh and Lynch 2018). This would initially result in a positive relationship between the MAF and the additive effect, where the positive effect alleles are more frequent at intermediate frequencies. Therefore, a negative relation between MAF and effect in populations under natural selection indicates purifying selection, whereas a positive relation indicates directional selection.

In populations under artificial selection, the estimated *s-*values should be interpreted differently than in populations under natural selection. This is because populations under artificial selection often have a smaller effective population size. In these populations, directional selection can result in a rapid increase in frequency of beneficial alleles until they reach fixation (Rowan et al. 2021), since the selection pressure is high on large-effect loci, resulting in a rapid increase in the allele frequency of these loci. Therefore, alleles with a large effect briefly remain at intermediate allele frequencies and occur, on average, at low MAF. The selection pressure on loci with small effects is relatively low, resulting in slower fixation and, on average, a higher MAF. Hence, in breeding populations under directional selection, a negative relationship between MAF and effect is expected. This negative relationship has been observed for important breeding goal traits in tilapia, dairy cattle, maize, and pigs (Joshi et al. 2021; Reynolds et al. 2022; Neshat et al. 2023; Palali Delen et al. 2023; Zhang et al. 2025). This negative relationship is in agreement with the results of our study, where we found an increasingly negative relationship between the additive effects and the MAF of the causal loci over 30 generations of directional selection (Supplementary Tables 1–4), which was correctly reflected in the estimated *s-*values.

Overall, we have seen that the optimal *s*-values became more negative when the number of generations of ongoing selection increased for a trait under directional selection (Fig. 10). However, when considering the $\Delta \mathrm{AI}C_{s}$ scores over these generations for a trait with heritability 0.3, $\Delta \mathrm{AI}C_{s}$ scores differed substantially from zero in 0%, 2%, 6%, 6%, 12%, 4%, 0% of the replicates for generation 1, 5, 10, 15, 20, 25, and 30, respectively (Supplementary Fig. 4). At first there was an increase in power up to generation 20, where selection was most often detected, after which there was a decrease in power, resulting in a decline in the detectivity of selection. As no new mutations were simulated during selection, more alleles reached fixation as the number of selected generations increased. This probably decreased the power to detect selection for a heritability of 0.3. For the lower heritabilities, the fixation of alleles occurred at a slower rate caused by the lower accuracy of selection, and therefore, we don’t see this pattern there.

### Bias of *s*-value estimations

For random selection, the MAF and effect were expected to be independent from each other, resulting in an expected optimal *s-*value of zero. Indeed, the regression coefficient for the MAF and the effect of the causal loci after 30 generations of selection after the burn-in was close to zero (0.02). However, the optimal *s-*values were, on average, estimated with SNPs to be around −0.2 to −0.3 for random selection. First, we investigated the optimal *s*-value using the causal loci as input, which resulted in an *s*-value of 0.1 (Fig. 11). This indicates that the expectation for an *s*-value of approximately zero is valid based on the causal loci only. Second, we investigated whether the negatively estimated *s*-values were a result of ascertainment bias, as the SNPs were sampled to have a uniform allele frequency distribution, while the causal loci had a U-shaped allele frequency distribution. This could result in not being able to explain the causal loci with low MAF accurately by the SNP, as is a common bias for SNP arrays (Nielsen 2004; Clark et al. 2005; Geibel et al. 2021). However, even with SNPs from a U-shaped allele frequency distribution, the optimal *s*-value was −0.3 for random selection with a heritability of 0.3 (Fig. 11), so the ascertainment bias was not the reason for finding an estimated *s*-value below zero. Overall, the ΔAIC scores for the optimal *s*-values for random selection reveal that for a maximum of 2% of the replicates, a value of $\Delta \mathrm{AI}C_{s}>10$ is found when comparing the optimum *s*-value to a value of zero (Supplementary Fig. 2). The *s*-values are thus estimated as slightly more negative based on SNPs than expected based on causal loci, but only rarely deviated significantly from zero.

For directional selection, the *s*-values were estimated to be −1 for both SNP arrays (uniform and U-shaped allele frequency distribution), whereas the *s*-value based on the causal loci was estimated to be −0.7. Like what was seen for random selection, the estimated *s-*values based on SNPs seem to be slightly more negative than the estimated *s*-values based on causal loci. This underestimation of the *s*-value could be due to LD between the causal loci and the SNPs, which is often incomplete, and not all causal loci can be completely explained by the SNP. Moreover, not all SNPs might be linked to a causal locus under selection. It is good to note that we have applied phenotypic selection, so the SNPs were not used for the selection. Therefore, it is expected that the causal loci reflect the true relationship between effect and MAF in the population. So, the *s*-values estimated from SNPs are more negative than from causal loci, thereby overestimating the actual selection signal at the causal variants.

### Comparing *s-*value estimation and $\hat{G}$

We have applied 2 methods to investigate selection history to evaluate their ability to detect selection in an animal breeding population under ongoing directional selection. The *s-*value estimation has been mostly applied to human populations to identify selection (Speed et al. 2017; Zeng et al. 2018; Speed et al. 2020; Zeng et al. 2021), and the interpretation of the *s-*values is slightly different for artificial selection as explained before. $\hat{G}$, on the other hand, is a method based on principles of genomic selection and has been applied to breeding populations (Beissinger et al. 2018).

When applying $\hat{G}$ in a population under directional selection, we have seen that for a selection interval of 10 generations, correct detectivity of selection occurred in 30%, 56%, and 98% of the replicates for a trait with a heritability of 0.05, 0.1, and 0.3, respectively. For each selection scenario this percentage increased when the selection interval became longer. The detectivity of selection for a heritability of 0.3 was nearly perfect for a selection interval of at least 5 generations. For a heritability of 0.1, selection was detected in at most 70% of the replicates, which occurred for a selection interval of 15 generations. For a heritability of 0.05 selection was identified at most for 38% of the replicates for a selection interval of 20 generations. In general, increasing the selection interval beyond 10 generations did not result in a large increase in detectivity of selection. This could be explained by the fact that marker effects are not constant over time. Ongoing selection changes allele frequencies, which in turn can alter LD patterns and the additive genetic variance. As a result, the estimated marker effects may change over generations (Wientjes et al. 2022). Extending the selection interval too far may, therefore, limit further improvement in detectivity, as ongoing allele frequency changes alter the underlying genetic architecture. From this, we can conclude that $\hat{G}$ detects selection reasonably well for low heritability traits, where only detectivity for a trait with a heritability of 0.05 was limited, although we do see small improvements when the selection interval is increased. It should be noted that the genotypes of the starting generation of this interval should be available; therefore, it might not always be possible to increase the selection interval further.

Another way to improve the detectivity of selection is potentially to increase the number of genotyped animals in the start and end generations, as shown by Mahmoud et al. (2023). Implementing this in practice would require that allele frequency data from 10 to 20 generations back in time would have to be available for these animals to potentially increase the detectivity of selection with $\hat{G}$ for a heritability of 0.05. Alternatively, initial allele frequencies could be inferred from pedigree data (Gengler et al. 2007), especially if the main parents of the investigated generations are genotyped. However, it has been described that this likely affects the detectivity of selection by reducing the power of the model (Beissinger et al. 2018).

Different from $\hat{G}$, the estimation of *s*-values uses only data from the most current generations. Estimating *s*-values as an indication of selection resulted in the correct identification of selection of 4%, 46%, and 100% for heritabilities of 0.05, 0.1, and 0.3, respectively, when the *s*-value was estimated using the last 5 generations of selection (Table 4). Increasing the number of generations, and thereby the number of genotyped animals, to which the method is applied, increased the detectivity of selection. Our study has shown that using the last 5 generations of the breeding population greatly increased the detectivity of selection compared with the last generation only, also for lower heritabilities.

The additive marker effects that are used to determine the relationship between the MAF and the effects can be more accurately estimated as the sample size increases (Daetwyler et al. 2008). So, enlarging the dataset further with more genotyped and phenotyped animals of one or more generations is expected to increase the correct identification of selection for the lower heritabilities more. However, it is good to realize that those marker effects can fluctuate over time as allele frequencies change under selection, thereby altering the LD patterns and the apparent size of marker effects (Goddard 2009). So, increasing the number of animals in the last generations can result in a more accurate estimation of the marker effects in the last generation, but increasing the number of animals across generations to estimate markers effects can help to estimate marker effects across generations that might be more related to the applied selection. Therefore, it is difficult to determine based on our results whether increasing the number of animals in the last generations or across generations contributes more strongly to improved detectivity. This should be considered when applying this method to empirical data.

In summary, considering both the strengths and weaknesses of each method, we can say that $\hat{G}$ is an intuitive method for breeders and the interpretation of positive or negative selection is straight forward, but the ability to reliably detect selection may be limited by the availability of the required data. However, it is good to realize that the allele frequency data of animals from 10 or more generations ago might not always be available, or not in the numbers required to detect selection. Nevertheless, gene content for ungenotyped individuals can be estimated from pedigree and genotype data of related animals, which can in turn be used to approximate allele frequencies (Gengler et al. 2007). $\hat{G}$ has shown to very accurately detect selection for traits with a moderate heritability (0.3). For lower heritabilities (0.05 and 0.1) the detectivity of selection is a bit lower but still substantial. Increasing the selection interval improved the detectivity, but increasing the number of genotyped animals could possibly improve the results further. In contrast to $\hat{G}$, the *s*-value method has no clear significance value and therefore the identification of selection is a bit more arbitrary. Furthermore, no distinction can be made between positive and negative selection, as both result in a negative value for *s* in populations under strong artificial selection. All in all, we have seen that both methods are capable of detecting selection in animal breeding populations, but there are different requirements for the dataset to reliably detect selection.

### Detection of indirect selection

Besides directly selected traits, we were interested in the potential to apply the methods to investigate the (indirect) selection history of new traits that might be interesting to add to the breeding goal. This is very relevant, because the focus of livestock breeding goals is currently shifting increasingly toward balanced breeding. Hence, novel traits related to robustness, resilience, welfare, environmental footprint, disease resistance, and social behavior have been or are currently being included in the breeding goal (Kanis et al. 2005; Merks et al. 2012; Harlizius et al. 2020). These new traits tend to have lower heritabilities because they are more complex and influenced by many genes and by the environment (Rydhmer and Lundeheim 2008; Turner et al. 2024). Due to genetic correlations with breeding goal traits, those new traits could have been under indirect selection even though the selection accuracy for these traits can be expected to be weak. Information on the selection history of a new trait can be used as an indication whether there are strong genetic correlations with other breeding goal traits, which is important to know before a trait is included in the breeding goal.

We simulated a trait under indirect selection by simulating a correlated trait (*h*^2^ = 0.05) of a directly selected trait (*h*^2^ = 0.1), with a genetic correlation of either 0.4 or 0.7. A genetic correlation of 0.4 is a more realistic value to be found between traits. The accuracy of indirect selection can be calculated as $r_{\mathrm{IHy}}=r_{x,y}r_{\mathrm{IHx}}$, where $r_{\mathrm{IH}}$ is the accuracy of the selected trait (*x*) and the response trait (*y*) and $r_{x,y}$ is the correlation between the traits (Mrode et al. 2023). For mass selection, the accuracy of the selected trait is equal to the square root of the heritability. Therefore, the accuracies of the indirectly selected traits were $\left(\sqrt{0.1}*0.4\approx \right)$ 0.13 for a correlation of 0.4 and $\left(\sqrt{0.1}*0.7\;\approx \right)$ 0.22 for a correlation of 0.7. Similarly, directional selection on the simulated trait with a heritability 0.05 had an accuracy of $\left(\sqrt{0.05}\approx \right)$ 0.22. We also aimed for to reach the same accuracy of selection with indirect selection as in one of the direct selection scenarios. That was why we simulated a rather high genetic correlation of 0.7 between the traits, while a lower genetic correlation of 0.4 is probably more realistic for actual breeding programs (Bergsma et al. 2013; Ye et al. 2018). We expected beforehand that the detectivity was related to the accuracy of selection, so that the detectivity was comparable for the trait under directional selection with a heritability of 0.05 and the trait under indirect selection with a correlation of 0.7. However, both $\hat{G}$ and the *s*-value estimation performed considerably poorer on the indirectly selected trait as on the directly selected trait.

A possible explanation for this difference in detectivity is the fixation of the large-effect loci (Beissinger et al. 2018). Under directional selection, causal loci fixate faster for traits with higher heritabilities, which was indeed seen in our simulation setup. In our case, selection on the indirect trait was simulated by direct selection on a trait with a heritability of 0.1, so the causal loci of the indirect trait fixed more rapidly than they would have if selection acted directly on a trait with a heritability of 0.05. It should be noted that the fixation of loci in the simulation likely exaggerates what occurs in real breeding programs, as the traits in the simulation shared strictly identical causal loci, the number of loci was finite, and only additive effects were considered. The estimated marker effects that were used for $\hat{G}$ were estimated in the last generation and therefore the loci that have fixed in generation 30 did not contribute to the analysis anymore. To determine the effect of the loss of these loci, additional analyses were done where the marker effects were estimated in the first generation of the selection interval (Supplementary Fig. 5 and Supplementary Table 5). This confirmed that using marker effects estimated in the first generation of the selection interval does indeed increase the detectivity for the trait under indirect selection, where the detectivity increased to at most 60% for indirect selection. Furthermore, an increase in the detectivity was also observed for direct selection when the marker effects were estimated in the first generation of the selection interval. This even resulted in a detectivity of 100% for both *h*^2^ = 0.05 and 0.1, for all selection intervals. Hence, estimating the marker effects of the starting generation could be valuable when both phenotype and genotype data are available from earlier generations.

The *s*-values that were estimated for indirect selection did not substantially differ from an *s*-value of zero based on the $\Delta \mathrm{AI}C_{s}$ scores. However, our results still showed some indications for selection. Looking at the last 5 generations of selection, the optimal *s*-value is −0.7 for the trait with a correlation of 0.7. The pattern of the replicates for this scenario is comparable to that for direct selection for a heritability of 0.05. Therefore, it is likely that the *s-*value estimation could be used to detect selection for indirect selected traits if more data would be available.

Overall, $\hat{G}$ is the preferred method to detect indirect selection, even though the results have shown that detectivity was initially limited using either $\hat{G}$ or the *s*-value estimation. However, using the estimated additive effects of the starting generation of the selection interval for $\hat{G}$ did improve the outcomes greatly. This means that genotype and phenotype data of this generation must be available.

## Conclusion

In conclusion, both $\hat{G}$ and the *s*-value estimation are able to detect direct selection in a simulated pig breeding population for traits with low to medium heritabilities. In general, higher heritabilities and a larger sample size (for *s*-value estimation) or a larger selection interval (for $\hat{G}$) result in better detectivity of selection. It depends on the available genotype- and phenotype-information and the trait of interest, which method would be best to apply. In general, $\hat{G}$ would be the preferred method to apply when allele frequency data from at least 10 generations back is available for a population, and phenotypes and genotypes from the most recent population, while the *s*-value estimation is the preferred method when only the latest generations of a population have been genotyped and phenotyped. The detectivity of indirect selection overall was limited, although $\hat{G}$ would be the preferred method for indirect selection. The detectivity of $\hat{G}$ could be increased, for both direct and indirect selection, if marker effects are estimated in the starting generation of the selection interval, for which genotypes and phenotypes of that generation are needed. Using these effects, $\hat{G}$ outperforms the *s-*value estimation in all cases.

## Supplementary Material

iyag136_Supplementary_Data

iyag136_Peer_Review_History

## Data Availability

All scripts used to generate the simulated data and perform the analyses are available at GSA FigShare (https://doi.org/10.25386/genetics.32247987). The files contain the parameter and seed files used for QMSim, a script to transform the QMSim output to files suitable to import into MoBPS, scripts used for the simulations in MoBPS, scripts to apply the Ghat R-package and bash files to estimate *s*-values. Supplemental material available at GENETICS online.
