# Supplementary material for: Methods to detect selection history in a population under ongoing directional selection
Source: Genetics. 2026 May 27;233(4):iyag136. doi: 10.1093/genetics/iyag136 (PMC13439694; doi:10.1093/genetics/iyag136)
Supplement: Supplementary file 1 [file iyag136_peer_review_history.pdf]

GENETICS

Methods to Detect Selection History in a Population under Ongoing Directional Selection

Anne Jansen, Mario Calus, and Yvonne Wientjes

NOTE: The reviews and decision letters are unedited and appear as submitted by the reviewers.
In extremely rare instances and as determined by a Senior Editor or the EIC, portions of a review may be redacted. If a review is signed, the reviewer has agreed to no longer remain anonymous.
The review history appears in chronological order.

Review Timeline:

Submission Date:	2025-11-10
Editorial Decision:	2026-02-03
Resubmission Received:	2026-04-02
Editorial Decision:	2026-04-20
Revision Received:	2026-05-11
Editorial Decision:	2026-05-12
Revision Received:	2026-05-13
Accepted:	2026-05-19

1st Editorial Decision: February 3, 2026

January 30, 2026

GENETICS-2025-308763
Selection History Models in a Population under Ongoing Directional Selection

Dear Dr. Jansen:

Two experts in the field have reviewed your manuscript, and I have read it as well. Both reviewers and I agree of the potential relevance of the work. While your manuscript is not currently acceptable for publication in GENETICS, we would welcome a substantially revised manuscript. Both reviewers have comments and concerns to be addressed in a revised manuscript. You can read their reviews at the end of this email.

Please, address major comments A and C from reviewer #1 and the main concern of reviewer number #2 related to the simulation of correlated traits, which is the main focus of the study. Whilst I can vaguely see that inference could potentially be made from comparing the directional and random selection simulations, there is a bit of a conceptual gap there that is hard for everyone to follow. Both reviewers mention this, one as main concern and the other as a comment. I think the impact of the paper would increase if this was addressed.

Please, also address the minor comments. They seem to me to be really minor.

We look forward to receiving your revised manuscript. Please let the editorial office know approximately how long you expect to need for revisions.

Upon resubmission, please include:
1. A clean version of your manuscript;
2. A marked version of your manuscript in which you highlight significant revisions carried out in response to the major points raised by the editor/reviewers (track changes is acceptable if preferred);
3. A detailed response to the editor's/reviewers' feedback and to the concerns listed above. Please reference line numbers in this response to aid the editor and reviewers.

Your paper will likely be sent back out for review.

Additionally, please ensure that your resubmission is formatted for GENETICS
https://academic.oup.com/genetics/pages/general-instructions

Follow this link to submit the revised manuscript: Link Not Available

Sincerely,

Albert Tenesa
Associate Editor
GENETICS

Approved by:
Anthony Long
Senior Editor
GENETICS

Reviewer #1 :

Title: Selection History Models in a Population under Ongoing Directional Selection
Reviewer Recommendation: Minor Revisions

1. General Summary
The authors investigate the utility of two statistical models, G-hat and BayesS, for detecting the history of selection on traits within a commercial animal breeding context. Using a simulated pig breeding program (QMSim), the study assesses whether these models can distinguish between traits under directional selection versus random drift, with a specific focus on low heritability traits and the impact of SNP array ascertainment bias.
The study finds that while both methods have merit, their sensitivity is highly dependent on heritability and the quantity of

available data (time intervals for Ghat, population size/variation for BayesS). A key theoretical contribution is the clarification that directional artificial selection produces a negative s-value signal similar to purifying selection in natural populations, albeit through different mechanisms (rapid fixation of large positive effects).

2. General Assessment
The manuscript addresses a relevant topic in modern quantitative genetics: "Balanced Breeding." As breeding goals expand to include welfare and environmental traits, understanding whether these new traits have been indirectly selected during historical production-focused breeding is valuable.
The simulation design is robust, and the distinction drawn between natural and artificial selection signatures is insightful. However, the practical utility of the methods for the stated goal-analyzing low heritability novel traits-seems limited by the results themselves, and the discussion could benefit from a more critical assessment of this limitation.

3. Major Comments
A. The "Low Heritability" Limitation vs. Study Objectives
The introduction sets a clear objective: to help breeders assess novel traits (e.g., behavior, resilience), which typically have low heritability. However, the results indicate that both Ghat and BayesS perform poorly at h^2 = 0.05.
For Ghat, detection was only ~30% in the best-case scenario for low heritability.
For BayesS, the standard deviation of estimates was high for low heritability, making significant detection difficult without massive sample sizes.
C. Simulation Parameters and Power
The study uses 10 replicates for the simulations. While 10 replicates can be sufficient for mean trends, it is on the lower side for assessing the variance of stochastic models like genetic drift. Given the high standard deviations observed in the s-value estimates (Figure 3 in the text), increasing replicates to 20-50 might provide a smoother distribution and higher confidence in the "false positive" rate of the random selection control group.
The Comparison of Methods: The comparison between Ghat and BayesS is slightly asymmetrical. Ghat requires temporal data (Generation X vs. Generation Y), while BayesS is a snapshot method (Generation X only). A "Decision Matrix" on when to use which method based on data availability would be beneficial for practitioners.

4. Minor Comments
Burn-in Period: The text mentions a 5-generation burn-in. Is this sufficient to establish Linkage Disequilibrium (LD) consistent with a commercial line before selection starts? Usually, longer burn-ins are preferred to stabilize LD, though the use of historical pig population parameters might mitigate this.
Abstract Clarity: The abstract mentions "indirect selection" as a motivation, but the results focus on direct selection on the trait. The authors should clarify in the discussion how these results extrapolate to indirect selection (genetic correlation), as that is the primary use case for novel traits.
I cannot currently access the figshare folder referenced in the Data Availability Statement. Access should be ensured before final acceptance.

Reviewer #2 :

Major
1. The major flaw is the conflict of the goal and the experimental design. The goal was to detect if a new trait outside of current breeding goal had been indirectly selected by correlated traits in the breeding goal (lines 10-11, 28-39). The author designed the indirectly selected traits as phenotypical selection + low heritability (lines 71-74, M&M), which did not match the definition they stated. This design was more of a traditional selection on an isolated low heritability trait, with no concern of correlation with any other trait. An accurate design should involve two correlated traits, one under random/directional selection and the other not, then compare the test results of both traits in all scenarios. If for any reason the authors do not want to change the design, then they should change the goal, or at least justify the reason of this design and discuss the flaw of it. However, retaining the current design may significantly diminish the overall impact and relevance of the paper.

Minor
1. Line 95, is it realistic to simulate uneven number of males and females per litter? If yes add citation, otherwise it's more reasonable to assume 1:1 sex ratio and select males with higher pressure than females.
2. Lines 118 and 130, it seems the author simulated in total 20K loci per chromosome, selected 2K as genes (casual loci), but they also said "from remaining segregating loci, 20K markers were selected...). 20K-2K=18K, not 20K. Check the numbers.
3. Lines 144-152, the author seemed to imply that the phenotypic variance was 1, because "additive genetic variance ... equal to ... heritability". Add some clarifications.
4. Lines 143-152 and many other places, "additive effects" "additive genetic values" are more of DGV. Clarify mendelian sampling effect was omitted or use DGV instead.
5. Line 162, define "the models".
6. Line 182, define r2.
7. Line 185, define G_perm hat bar.

8. Lines 193-246 s-value estimation section, it seems the author tested 21 s-values = -1, -0.9, -0.8 ... 1. Clarify it here.
9. Line 257-259, add citation.
10. Line 268 and many other places, "effect size" is confusing. Can replace with "effect" directly or "magnitude of effect".
11. Line 282. Change "no development" to "little deviation" and add a P value for test against 0. Otherwise, it seems from line 282 that, with higher h2, the random selections still had more negative mean BV. Also, your figure 9 kind of proved that there was a "selection", and you also stated that there was a genetic drift.
12. Figure 4, use a different colour group for random selection scenarios. Explain the difference between lines and shades around lines.
13. Lines 286-300, the G hat values aren't shown. It will be good to see if they are negative or positive, and if their signs are consistent with those of s estimates.
14. Line 308, did that mean s = -1 was AIC_min and the log likelihood was used as the max log likelihood? If yes clarify it. Also discuss the implication that s=-1 in most cases had the highest likelihood in the Discussion.
15. Lines 312 and 317, define "compared to". Is it actually "at"? Also, the authors can be more explicit about the table 3 results, indicating the more the replicates exceeding a delta AIC > 10, the more likely s=0 is the wrong model, the stronger the evidence about selection. The whole s value method is already confusing, and the results are even more confusing in general.
16. Line 322, table 3 title. Similar to above, add more interpretations.
17. Figure 6 and 7 titles wrong. It's the difference of log likelihood values, not the absolute log likelihood values.
18. Figures 6 and 7, why is that the figures and corresponding results and discussions only showed s test value range of -1 to 0? In Appendix there were full range of s test results. Why did the authors decide the positive s values should be ignored? Is it due to low log likelihood values? Clarify in the Results the reason to ignore positive s values and explain in Discussion the implication.
19. Line 336, add "vertical lines" after "red".
20. Lines 378-384, show adj-R2 of the regression fit, and coefficient SD and P-values. It's likely to be insignificant for at least some h2. And that changes the narrative.
21. Line 414, 0.3 for production traits are quite small compared to production traits in, say, dairy cows. Is liveweight also measured? Is the h2 higher?
22. Lines 432-436, see the major change section comment.
23. Lines 462-465, authors can check the regression in earlier generations and see how slope changes across generations to prove this assumption, instead of only checking the last generation's slope, since gen 30 is a relative arbitrary number.
24. Lines 570-576, move "However, it is good to ... required to detect selection." before "G hat has shown to be very accurately...".
25. Lines 567-581, do you think the tested best s values can be applied to real genetic evaluation pipeline?
26. Lines 582-590, the conclusion is accurate but did not address the implication related to the goals.

English writing

- The English writing quality is not too good. The main problem is the tense. Sometimes there are both past and present tense in the same sentence. It's suggested to use past tense in abstract, introduction, M&M and results. The discussion and conclusion can use different tenses depending on the context. For example, if it refers to the experiment and the results, then use past tense.
- The other problem is mis- or mixed use of singular and plural nouns and verbs. E.g. line 339 should be "the thick lines are the average of these replicates" since it's referring to all 9 sub figures.
- Line 23 and other places, change "detection" to "the detectivity" or "the effectiveness of detection on selection".
- Line 23 and other places, change "model" to "method" when referring to s-value or G hat methods

Associate Editor Comments: Apologies for the delay it was hard to identify reviewers.

General reply to editor's and reviewers' comments:

We thank both reviewers and the associated editor for their comments. Please find our reply to each of the comments preceded with "AU:" below.

Associated editor comments

Two experts in the field have reviewed your manuscript, and I have read it as well. Both reviewers and I agree of the potential relevance of the work. While your manuscript is not currently acceptable for publication in GENETICS, we would welcome a substantially revised manuscript. Both reviewers have comments and concerns to be addressed in a revised manuscript. You can read their reviews at the end of this email.

Please, address major comments A and C from reviewer #1 and the main concern of reviewer number #2 related to the simulation of correlated traits, which is the main focus of the study. Whilst I can vaguely see that inference could potentially be made from comparing the directional and random selection simulations, there is a bit of a conceptual gap there that is hard for everyone to follow. Both reviewers mention this, one as main concern and the other as a comment. I think the impact of the paper would increase if this was addressed.

Please, also address the minor comments. They seem to me to be really minor.
AU: Thank you for the comments and good to read that our work has potential relevance. We have addressed the comments of the reviewers, with the main addition being the simulation of an indirect selection scenario. Please see our response to the reviewer comments below.

Reviewer #1 :

Title: Selection History Models in a Population under Ongoing Directional Selection
Reviewer Recommendation: Minor Revisions

1. General Summary
The authors investigate the utility of two statistical models, G-hat and BayesS, for detecting the history of selection on traits within a commercial animal breeding context. Using a simulated pig breeding program (QMSim), the study assesses whether these models can distinguish between traits under directional selection versus random drift, with a specific focus on low heritability traits and the impact of SNP array ascertainment bias.
The study finds that while both methods have merit, their sensitivity is highly dependent on heritability and the quantity of available data (time intervals for Ghat, population size/variation for BayesS). A key theoretical contribution is the clarification that directional artificial selection produces a negative s-value signal similar to purifying selection in natural populations, albeit through different mechanisms (rapid fixation of large positive effects).

2. General Assessment
The manuscript addresses a relevant topic in modern quantitative genetics: "Balanced Breeding." As breeding goals expand to include welfare and environmental traits, understanding whether these new traits have been indirectly selected during historical production-focused breeding is valuable. The simulation design is robust, and the distinction drawn between natural and artificial selection

signatures is insightful. However, the practical utility of the methods for the stated goal-analyzing low heritability novel traits-seems limited by the results themselves, and the discussion could benefit from a more critical assessment of this limitation.

AU: Thank you for the positive comments. Please see our response below to the comment about the simulation of low heritability novel traits.

3. Major Comments

A. The "Low Heritability" Limitation vs. Study Objectives

The introduction sets a clear objective: to help breeders assess novel traits (e.g., behavior, resilience), which typically have low heritability. However, the results indicate that both Ghat and BayesS perform poorly at h^2 = 0.05.

For Ghat, detection was only ~30% in the best-case scenario for low heritability.

For BayesS, the standard deviation of estimates was high for low heritability, making significant detection difficult without massive sample sizes.

AU: We agree that our results show that it is difficult to detect selection for traits with low heritabilities when sample sizes are small. As we have described in the discussion and conclusion section, it is possible to detect selection for lower heritabilities, but enough data is needed, and this might not always be available. We have emphasized this more in the Discussion (Line 595-597, 632-636). Furthermore, we have now included additional analysis for Ghat, showing that the detectivity of selection can greatly increase when SNP-effects are not estimated in the last generation of the interval, but at the starting generation of the interval (Line 672-688, 714-719).

C. Simulation Parameters and Power

The study uses 10 replicates for the simulations. While 10 replicates can be sufficient for mean trends, it is on the lower side for assessing the variance of stochastic models like genetic drift. Given the high standard deviations observed in the s-value estimates (Figure 3 in the text), increasing replicates to 20-50 might provide a smoother distribution and higher confidence in the "false positive" rate of the random selection control group.

AU: We did create the base population for 50 replicates, after which we have simulated directional and random selection with three different heritabilities, all with 50 replicates. We have rephrased this in the manuscript to make this clearer (Line 114). We have further clarified this in Table 1, which gives an overview of the selection strategies (Line 178-179).

The Comparison of Methods: The comparison between Ghat and BayesS is slightly asymmetrical. Ghat requires temporal data (Generation X vs. Generation Y), while BayesS is a snapshot method (Generation X only). A "Decision Matrix" on when to use which method based on data availability would be beneficial for practitioners.

AU: We agree that Ghat and the s-value estimation cannot be compared directly because of the different input data. We did include some general suggestions based on our findings, which we have now further clarified and summarized in the conclusion (Line 712-719). We don't think that a decision matrix that can be drawn based on our study, will add a lot of value to this description and have decided not to include this.

4. Minor Comments

Burn-in Period: The text mentions a 5-generation burn-in. Is this sufficient to establish Linkage

Disequilibrium (LD) consistent with a commercial line before selection starts? Usually, longer burn-ins are preferred to stabilize LD, though the use of historical pig population parameters might mitigate this.
AU: The historical populations consisted of 5,000 generations of random mating, which were simulated to generate an LD structure that is comparable to pigs from breeding lines, which is shown in Figure 3. During the burn-in period of 5 generations the selection strategy of that replicate is applied, to establish population structure under this scenario. We have clarified this in the manuscript (Line 97, 112)

Abstract Clarity: The abstract mentions "indirect selection" as a motivation, but the results focus on direct selection on the trait. The authors should clarify in the discussion how these results extrapolate to indirect selection (genetic correlation), as that is the primary use case for novel traits.
AU: Thank you for this comment. We agree that our motivation did not completely align with only direct selection scenarios. We have now expanded the objective of the paper, to cover both direct and indirect selection (Line 82-83). Moreover, we have now included some indirect selection scenarios, with two different genetic correlations between traits to demonstrate the detectivity of the models for indirectly selected traits (Abstract: Line 18-19, 21-23, 25-30; Methods: Line 105, 160-170, 175-179; Results: Line 328-341, 386-401; Discussion: Line 463-465, 468-469, 475-476, 646-704; Conclusion: Line 714-719; Appendix: Line 888-891, 905-907).

I cannot currently access the figshare folder referenced in the Data Availability Statement. Access should be ensured before final acceptance.
AU: We will reupload the scripts, including the new scripts, into the Figshare folder during the submission process.

Reviewer #2 :

Major
1. The major flaw is the conflict of the goal and the experimental design. The goal was to detect if a new trait outside of current breeding goal had been indirectly selected by correlated traits in the breeding goal (lines 10-11, 28-39). The author designed the indirectly selected traits as phenotypical selection + low heritability (lines 71-74, M&M), which did not match the definition they stated. This design was more of a traditional selection on an isolated low heritability trait, with no concern of correlation with any other trait. An accurate design should involve two correlated traits, one under random/directional selection and the other not, then compare the test results of both traits in all scenarios. If for any reason the authors do not want to change the design, then they should change the goal, or at least justify the reason of this design and discuss the flaw of it. However, retaining the current design may significantly diminish the overall impact and relevance of the paper.
AU: Thank you for this comment. We agree that our motivation did not completely align with only direct selection scenarios. We have now expanded the objective of the paper, to cover both direct and indirect selection (Line 82-83). Moreover, we have now included some indirect selection scenarios, with two different genetic correlations between traits to demonstrate the detectivity of the models for indirectly selected traits (Abstract: Line 18-19, 21-23, 25-30; Methods: Line 105, 160-170, 175-179; Results: Line 328-341, 386-401; Discussion: Line 463-465, 468-469, 475-476, 646-704; Conclusion: Line 714-719; Appendix: Line 888-891, 905-907).

Minor

1. Line 95, is it realistic to simulate uneven number of males and females per litter? If yes add citation, otherwise it's more reasonable to assume 1:1 sex ratio and select males with higher pressure than females.

AU: The unequal sex ratio represents the unequal number of selection candidates per sex, we have now clarified this (Line 101-103).

2. Lines 118 and 130, it seems the author simulated in total 20K loci per chromosome, selected 2K as genes (casual loci), but they also said "from remaining segregating loci, 20K markers were selected...). 20K-2K=18K, not 20K. Check the numbers.

AU: There are 20K loci per chromosome, and there are 18 chromosomes. From the segregating loci, 20K loci were sampled in total (not per chromosome). We have clarified this in the methods (Line 139-140).

3. Lines 144-152, the author seemed to imply that the phenotypic variance was 1, because "additive genetic variance ... equal to ... heritability". Add some clarifications.

AU: We have added clarifications on the variances as suggested (Line 154-159).

4. Lines 143-152 and many other places, "additive effects" "additive genetic values" are more of DGV. Clarify mendelian sampling effect was omitted or use DGV instead.

AU: We have now clearly indicated that the Mendelian sampling variation was represented by random segregation of chromosomes (Line 128-129). We have now used 'additive effects' to refer to the effects of causal loci, and 'additive genetic values' to refer to the breeding value of an animal (Line 283, 285, 443, 445, 457)

5. Line 162, define "the models".

AU: This sentence was removed.

6. Line 182, define r2.

AU: We have added a clarification in the Methods (Line 195-196).

7. Line 185, define G_perm hat bar.

AU: We have added the definition (Line 198-199).

8. Lines 193-246 s-value estimation section, it seems the author tested 21 s-values = -1, -0.9, -0.8 ... 1. Clarify it here.

AU: We have added clarifications (Line 241-242).

9. Line 257-259, add citation.

AU: Done (Line 274-275).

10. Line 268 and many other places, "effect size" is confusing. Can replace with "effect" directly or "magnitude of effect".

AU: We have changed "effect size" to "effect" (Line 221, 283, 445, 497, 502, 506, 507, 520, 539, 541, 567)

11. Line 282. Change "no development" to "little deviation" and add a P value for test against 0. Otherwise, it seems from line 282 that, with higher h2, the random selections still had more negative mean BV. Also, your figure 9 kind of proved that there was a "selection", and you also stated that there was a genetic drift.

AU: We have changed "no development" to "little deviation" and included a one-sample t-test to test if the mean breeding value in the last generation differs from 0 for the random selection scenarios. We realized that Figure 4 originally showed the average breeding value over the animals in one replicate, which was not specified in the supporting text. The figure does now include all 50 replicates, and the BVs for random selection do not differ from 0 (Line 292-306).

Regarding Figure 9 (now figure 11), the causal loci resulted in a mean s-value that deviated slightly from 0, this can be explained by genetic drift (Line 433-434).

12. Figure 4, use a different colour group for random selection scenarios. Explain the difference between lines and shades around lines.

AU: We have added the ribbons around the lines to the description of the figure (Line 303-306). The random selection scenarios overlap mostly and therefore are hard to distinguish in any colour, the ribbons should now be clearer.

13. Lines 286-300, the G hat values aren't shown. It will be good to see if they are negative or positive, and if their signs are consistent with those of s estimates.

AU: All significant Ghat values are positive, which is expected for the directional positive selection that was applied. This is opposite of the s-value estimates, because they are not directly comparable. Ghat uses the change in allele frequency and the estimated marker effects. As the positive effects increase and negative effects decrease in allele frequency, we expect a positive correlation between the estimated marker effects and the change in allele frequency. For the s-value estimation, the relation between the absolute effect and the current allele frequency is modelled. Therefore, Ghat values and s-values are not expected to be directly comparable.

We have now described this for Ghat (Line 315-317). Regarding the negative s-values, this was further discussed in the Discussion (Line 498-509).

14. Line 308, did that mean s = -1 was AIC_min and the log likelihood was used as the max log likelihood? If yes clarify it. Also discuss the implication that s=-1 in most cases had the highest likelihood in the Discussion.

AU: The s-value of -1 indeed had on average the highest likelihood. Therefore, the s = -1 was for a number of replicates indeed the AIC_min value. The delta AIC scores were calculated for all 50 replicates separately, so the AIC_min value varied for the replicates (Line 344-346).

15. Lines 312 and 317, define "compared to". Is it actually "at"? Also, the authors can be more explicit about the table 3 results, indicating the more the replicates exceeding a delta AIC > 10, the more likely s=0 is the wrong model, the stronger the evidence about selection. The whole s value method is already confusing, and the results are even more confusing in general.

AU: We have clarified the section (Line 349-352, 353-354, 357) and implemented the suggestion to be more explicit about the results (Line 354-355, 362-363).

16. Line 322, table 3 title. Similar to above, add more interpretations.

AU: We have changed the title of table 3 (Line 374-375).

17. Figure 6 and 7 titles wrong. It's the difference of log likelihood values, not the absolute log likelihood values.

AU: The titles are changed (Line 377-379, 382-383, 420-423, 436-438).

18. Figures 6 and 7, why is that the figures and corresponding results and discussions only showed s test value range of -1 to 0? In Appendix there were full range of s test results. Why did the authors decide the positive s values should be ignored? Is it due to low log likelihood values? Clarify in the Results the reason to ignore positive s values and explain in Discussion the implication.

AU: Figures 6 and 7 show results for all tested s-values from -1 to 1 for all 50 replicates. We have clarified this in the manuscript (Line 344-346, 364). For any of the replicates where an s-value > 0 was identified as the best s-value (highest likelihood for that replicate), the delta AICs score did not reach a value >10 for directional selection.

19. Line 336, add "vertical lines" after "red".

AU: Done (Line 380).

20. Lines 378-384, show adj-R2 of the regression fit, and coefficient SD and P-values. It's likely to be insignificant for at least some h2. And that changes the narrative.
AU: We have now included the adjusted R2 values, coefficient SD and p-values and interpretation of these values (Line 447-453).
21. Line 414, 0.3 for production traits are quite small compared to production traits in, say, dairy cows. Is liveweight also measured? Is the h2 higher?
AU: In this section we are specifically describing comparable traits in pigs, because our aim was to simulate a pig breeding program. We have therefore clarified this in the manuscript (Line 489).
22. Lines 432-436, see the major change section comment.
AU: We have changed this section and clarified this in the discussion (Line 646-704).
23. Lines 462-465, authors can check the regression in earlier generations and see how slope changes across generations to prove this assumption, instead of only checking the last generation's slope, since gen 30 is a relative arbitrary number.
AU: We have now checked the regression in earlier generations as well (generation 1, 5, 10, 15, 20, 25, and 30), this is added to the manuscript (Methods: Line 283, 286; Results: Line 445-446; Discussion: Line 523-524; Appendix: Line 896-904).
24. Lines 570-576, move "However, it is good to ... required to detect selection." before "G hat has shown to be very accurately...".
AU: Done (Line 632-634).
25. Lines 567-581, do you think the tested best s values can be applied to real genetic evaluation pipeline?
AU: In our study we did not focus on genomic evaluation, and we don't think we can indicate if this could be applied based on what we have studied.
26. Lines 582-590, the conclusion is accurate but did not address the implication related to the goals.
AU: The conclusion does now also address indirect selection (Line 714-719).

English writing
• The English writing quality is not too good. The main problem is the tense. Sometimes there are both past and present tense in the same sentence. It's suggested to use past tense in abstract, introduction, M&M and results. The discussion and conclusion can use different tenses depending on the context. For example, if it refers to the experiment and the results, then use past tense.
AU: We have corrected the tense throughout the manuscript (Line 133, 134, 143, 183, 185, 192, 201-203, 232, 236, 259, 346, 454)
• The other problem is mis- or mixed use of singular and plural nouns and verbs. E.g. line 339 should be "the thick lines are the average of these replicates" since it's referring to all 9 sub figures.
AU: We changed this at several locations (Line 139, 379, 384-385, 422-423, 440-441).
• Line 23 and other places, change "detection" to "the detectivity" or "the effectiveness of detection on selection".
AU: We have changed this throughout the manuscript (Line 25, 26, 328, 331, 467, 484, 532, 579, 582, 591, 593, 598, 602, 605, 612, 613, 627, 638, 639, 709).
• Line 23 and other places, change "model" to "method" when referring to s-value or G hat methods
AU: We have changed this throughout the manuscript (Line 1, 12, 23, 27, 46, 69, 72, 79, 180, 181, 185, 207, 290, 308, 309, 343, 461, 571, 575, 611, 627, 629, 630, 644, 710, 711, 713).

Associate Editor Comments: Apologies for the delay it was hard to identify reviewers.

April 20, 2026
RE: GENETICS-2026-309326

Dear Dr. Jansen:

I am pleased to accept your manuscript titled "Methods to Detect Selection History in a Population under Ongoing Directional Selection" for publication in GENETICS, pending minor revision.

Please submit your revision along with a brief description of how you modified the manuscript in response to the reviewers' concerns and suggestions (which can be viewed at the bottom of this email). Most important are that if you agree that you add in the discussion the three points the reviewer raises, please do also take care of the small formatting/wording issues pointed out by the reviewer. I expect you should be able to submit a revised manuscript within 30 days. A suitably revised manuscript will be acceptable for publication; I don't expect to send it out for review.

Prior to uploading your revised manuscript please format it according to GENETICS style and ensure you have all required elements. Author Guidelines are at https://academic.oup.com/genetics/pages/author-guidelines#section-12. These guidelines have been updated with new requirements and your careful attention is required to avoid delays at the source file stage post-acceptance.

***EDITORS REMOVE IF THIS IS PRESENT/DOESN"T APPLY
Please include a Data Availability Statement in your article under the heading "Data availability," before the Acknowledgements section. See https://academic.oup.com/genetics/pages/author-guidelines#section-12-11 for details. The DAS should include the accession numbers or DOIs of any data you have placed in public repositories, describe supplemental material, include applicable IRB numbers, and may include specifications for how to properly acknowledge or cite the data.

When revising the ms., please make an effort to shorten it, because that almost always improves a manuscript. We urge authors to heed the advice of Strunk and White: "omit needless words"1. Follow this link to submit the revised manuscript: Link Not Available

Thank you for submitting this story to Genetics.

Sincerely,

Albert Tenesa
Associate Editor
GENETICS

Approved by:
Anthony Long
Senior Editor
GENETICS

Reviewer comments:
Reviewer #2 :

Comments:
The authors answered most questions well, and made revisions to the suggestions. I appreciate their effort. In general I am happy with the new draft with some minor revisions.

The added indirect trait simulation is desirable, but the heritability of index and indirect traits were 0.1 and 0.05. This can be realistic if the index trait is number of piglet born alive or birth weight, and the indirect trait to be piglet survival. In reality, a lot of growth, carcass and feed efficiency traits have moderate heritability, and indirect traits such temperament, feeding/social behavior and skin damage can have moderate to high heritability. Therefore, this paper presented a worst case scenario, and

demonstrated limited detectivity. It will be good to indicate in the discussion that in practice the detectivity can be better.

The correlation between the direct and indirect traits was simulated as 0.4 and 0.7. In reality, most correlations are between -0.3 and 0.4, so 0.7 in this draft is too high.

The authors also tried using gen1 instead of gen30's SNP effect in the G hat method, resulting in a better detectivity in general. This can be useful in research populations when >=5 generations of genotypes are available.

Minor changes:
1. Appendix VI table, effect 1 and 2 are from gen 1 and gen 30/29.
2. Tables 2 and 3, the column header for gen. 10-30 etc. is missing.
3. Figure 5 title says "different number of generations of selection" but it should be "different selection intervals from the last generation". Same for the x axis label.
4. Figure 6 and Appendix VI plots x axis label. See above.

Associate Editor comments:

General reply to editor's and reviewers' comments:

We thank the reviewer and the associated editor for their comments. Please find our reply to each of the comments preceded with "AU:" below.

Associated editor comments
I am pleased to accept your manuscript titled "Methods to Detect Selection History in a Population under Ongoing Directional Selection" for publication in GENETICS, pending minor revision.

Please submit your revision along with a brief description of how you modified the manuscript in response to the reviewers' concerns and suggestions (which can be viewed at the bottom of this email). Most important are that if you agree that you add in the discussion the three points the reviewer raises, please do also take care of the small formatting/wording issues pointed out by the reviewer. I expect you should be able to submit a revised manuscript within 30 days. A suitably revised manuscript will be acceptable for publication; I don't expect to send it out for review.
AU: Thank you for the comments and we are pleased to hear that our manuscript is acceptable for publication, pending the revisions. We have addressed the comments of the reviewer, please see our response to these comments below.

Reviewer #2 :
Comments:

The authors answered most questions well, and made revisions to the suggestions. I appreciate their effort. In general I am happy with the new draft with some minor revisions.
AU: Thank you for the positive comments. Please see our response below, where we have addressed the comments.

The added indirect trait simulation is desirable, but the heritability of index and indirect traits were 0.1 and 0.05. This can be realistic if the index trait is number of piglet born alive or birth weight, and the indirect trait to be piglet survival. In reality, a lot of growth, carcass and feed efficiency traits have moderate heritability, and indirect traits such temperament, feeding/social behaviour and skin damage can have moderate to high heritability. Therefore, this paper presented a worst-case scenario and demonstrated limited detectivity. It will be good to indicate in the discussion that in practice the detectivity can be better.
AU: The reviewer is right to mention that the chosen heritabilities are on the lower side. We discussed heritabilities of varying pig traits in the discussion (Line 426-433). We have now acknowledged that we simulated a low heritability trait (comparable to a reproductive trait), but that the detectivity would increase if the heritability was higher (more comparable to growth traits). Therefore, in real populations detectivity could be better (Line 439-443).

The correlation between the direct and indirect traits was simulated as 0.4 and 0.7. In reality, most correlations are between -0.3 and 0.4, so 0.7 in this draft is too high.
AU: We agree that a genetic correlation of 0.7 is high. In our study, we aimed to simulate a trait under indirect selection with a similar selection accuracy to one of the directly selected traits. The correlation of 0.4 is more realistic We have further clarified this in the discussion (Line 608-609, 615-622).

The authors also tried using gen1 instead of gen30's SNP effect in the G hat method, resulting in a better detectivity in general. This can be useful in research populations when >=5 generations of genotypes are available.

AU: The detectivity indeed increased when the effects were estimated in earlier generations, which is very useful when genotypes and phenotypes are available from this generation. We have emphasized this in the Discussion (Line 641-643).

Minor changes:

1. Appendix VI table, effect 1 and 2 are from gen 1 and gen 30/29.

AU: This has been added (Supplementary File S1; Line 46-49).

2. Tables 2 and 3, the column header for gen. 10-30 etc. is missing.

AU: This has been added to Table 2 and 3 (Line 875; 878), as well as in Table 4 (Line 881) and Table E (Supplementary File S1; Line 48).

3. Figure 5 title says "different number of generations of selection" but it should be "different selection intervals from the last generation". Same for the x axis label.

AU: This was changed (Line 843)

4. Figure 6 and Appendix VI plots x axis label. See above.

AU: This was changed for Figure 6 (Line 846) and Figure E (Supplementary File S1; Line 36, 40)

May 12, 2026
RE: GENETICS-2026-309326R1

Dear Dr. Jansen:

I am pleased to accept your manuscript titled "Methods to Detect Selection History in a Population under Ongoing Directional Selection" for publication in GENETICS, pending minor revision.

Please submit your revision along with a brief description of how you modified the manuscript in response to the reviewers' concerns and suggestions (which can be viewed at the bottom of this email. One of the reviewers minor comments don't seem to be changed (3. Figure 5 title says "different number of generations of selection" but it should be "different selection intervals from the last generation". Same for the x axis label.
AU: This was changed (Line 843)) . Line 843 is a reference line and Fig 5 legend doesn't have the text suggested by reviewer. I expect you should be able to submit a revised manuscript within 30 days. A suitably revised manuscript will be acceptable for publication; I don't expect to send it out for review.

Prior to uploading your revised manuscript please format it according to GENETICS style and ensure you have all required elements. Author Guidelines are at https://academic.oup.com/genetics/pages/author-guidelines#section-12. These guidelines have been updated with new requirements and your careful attention is required to avoid delays at the source file stage post-acceptance.

***EDITORS REMOVE IF THIS IS PRESENT/DOESN"T APPLY
Please include a Data Availability Statement in your article under the heading "Data availability," before the Acknowledgements section. See https://academic.oup.com/genetics/pages/author-guidelines#section-12-11 for details. The DAS should include the accession numbers or DOIs of any data you have placed in public repositories, describe supplemental material, include applicable IRB numbers, and may include specifications for how to properly acknowledge or cite the data.

When revising the ms., please make an effort to shorten it, because that almost always improves a manuscript. We urge authors to heed the advice of Strunk and White: "omit needless words"1. Follow this link to submit the revised manuscript: Link Not Available

Thank you for submitting this story to Genetics.

Sincerely,

Albert Tenesa
Associate Editor
GENETICS

Approved by:
Anthony Long
Senior Editor
GENETICS

Reviewer comments:

Associate Editor comments:

General reply to editor's and reviewers' comments:

We thank the associated editor for their comment. Please find our reply to each of the comments preceded with "AU:" below.

Associated editor comments

I am pleased to accept your manuscript titled "Methods to Detect Selection History in a Population under Ongoing Directional Selection" for publication in GENETICS, pending minor revision.

Please submit your revision along with a brief description of how you modified the manuscript in response to the reviewers' concerns and suggestions (which can be viewed at the bottom of this email.

One of the reviewers minor comments don't seem to be changed (3. Figure 5 title says "different number of generations of selection" but it should be "different selection intervals from the last generation". Same for the x axis label.

AU: This was changed (Line 843)). Line 843 is a reference line and Fig 5 legend doesn't have the text suggested by reviewer. I expect you should be able to submit a revised manuscript within 30 days. A suitably revised manuscript will be acceptable for publication; I don't expect to send it out for review.

AU: We are sorry to hear that there is a mishap regarding Figure 5. We have checked the files that were uploaded. In the file 'Merged File containing manuscript text and 12 Figure files', the revision is present on Line 843, where we changed 'different number of generations of selection' into 'different selection intervals relative to the last generation'. We will reupload the files and thereby hope that any unclearness is resolved.

May 19, 2026
RE: GENETICS-2026-309326R2

Ms. Anne C.M. Jansen
Wageningen University & Research
Animal Breeding and Genomics
Droevendaalsesteeg 1
Wageningen
Netherlands

Dear Dr. Jansen:

Congratulations, your manuscript titled "Methods to Detect Selection History in a Population under Ongoing Directional Selection" is accepted for publication in GENETICS! Many thanks for submitting your research to the journal.

To Proceed to Publication:
1. Format your article according to GENETICS style: https://academic.oup.com/genetics/pages/author-guidelines

2. Ensure that you comply with data and community resource citation guidelines:
https://academic.oup.com/genetics/pages/author-guidelines#section-5-9-2

3. Upload your final files at https://genetics.msubmit.net

4. Add oupsupport@scipris.com and genetics.oup@novatechset.com (or the domains @scipris.com and @novatechset.com) to your email program's "safe senders" list. You will be contacted by both at various points during the production process.

Notes:
- Your currently-accepted manuscript (unedited, as submitted, reviewed, and accepted) will be published at GENETICS and deposited into PubMed as an Advance Access article. Notify sourcefiles@thegsajournals.org before signing your license if you do not wish to publish your article via Advance Access.

- We invite you to submit an original color figure related to your paper for consideration as cover art. Please email your submission to the editorial office or upload it with your final files. You can submit a small-sized image for evaluation, and if selected, the final image must be a TIFF file 2513px wide by 3263px high (8.375 by 10.875 inches; resolution of 600ppi). Please avoid graphs and small type.

- After files are sent to Oxford University Press we use SciPris to manage article licensing and payment. If you do not have a SciPris account, you will receive an email from no-reply@scipris.com to sign up to use Oxford University Press' author portal. After logging in, follow the online instructions to sign your license and arrange any payment due.

If you have any questions or encounter any problems while uploading your accepted manuscript files, please email the editorial office at sourcefiles@thegsajournals.org.

Sincerely,

Albert Tenesa
Associate Editor
GENETICS

Approved by:
Anthony Long
Senior Editor
GENETICS
