# Supplementary material for: Methods to detect selection history in a population under ongoing directional selection
Source: Genetics. 2026 May 27;233(4):iyag136. doi: 10.1093/genetics/iyag136 (PMC13439694; doi:10.1093/genetics/iyag136)
Supplement: Supplementary file 2 [file iyag136_supplementary_data.pdf]

# Methods to Detect Selection History in a Population under Ongoing Directional Selection

Anne C.M. Jansen[1*], Mario P.L. Calus[1], Yvonne C.J. Wientjes[1]

[1]Animal Breeding and Genomics, Wageningen University and Research, 6700 AH, Wageningen, The Netherlands

*Corresponding author: anne.jansen@wur.nl

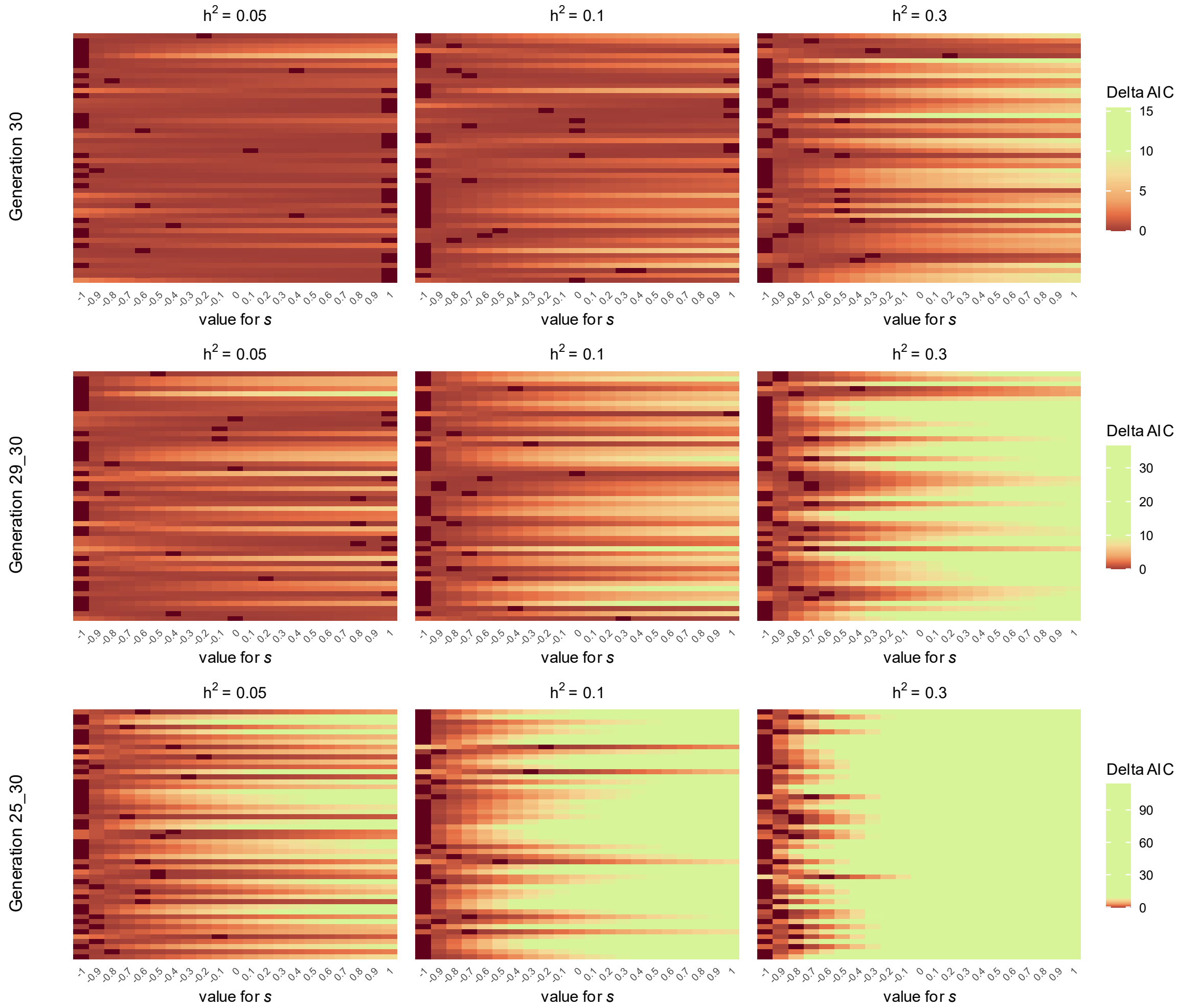


Supplementary Figure 1. $\Delta AIC_s$ scores for $s$-value estimation on directional selection on a trait with a heritability of 0.05, 0.1 and 0.3. The results are given for generation 30, 29-30, and 25-30, the rows represent the 50 replicates for each selection scenario.

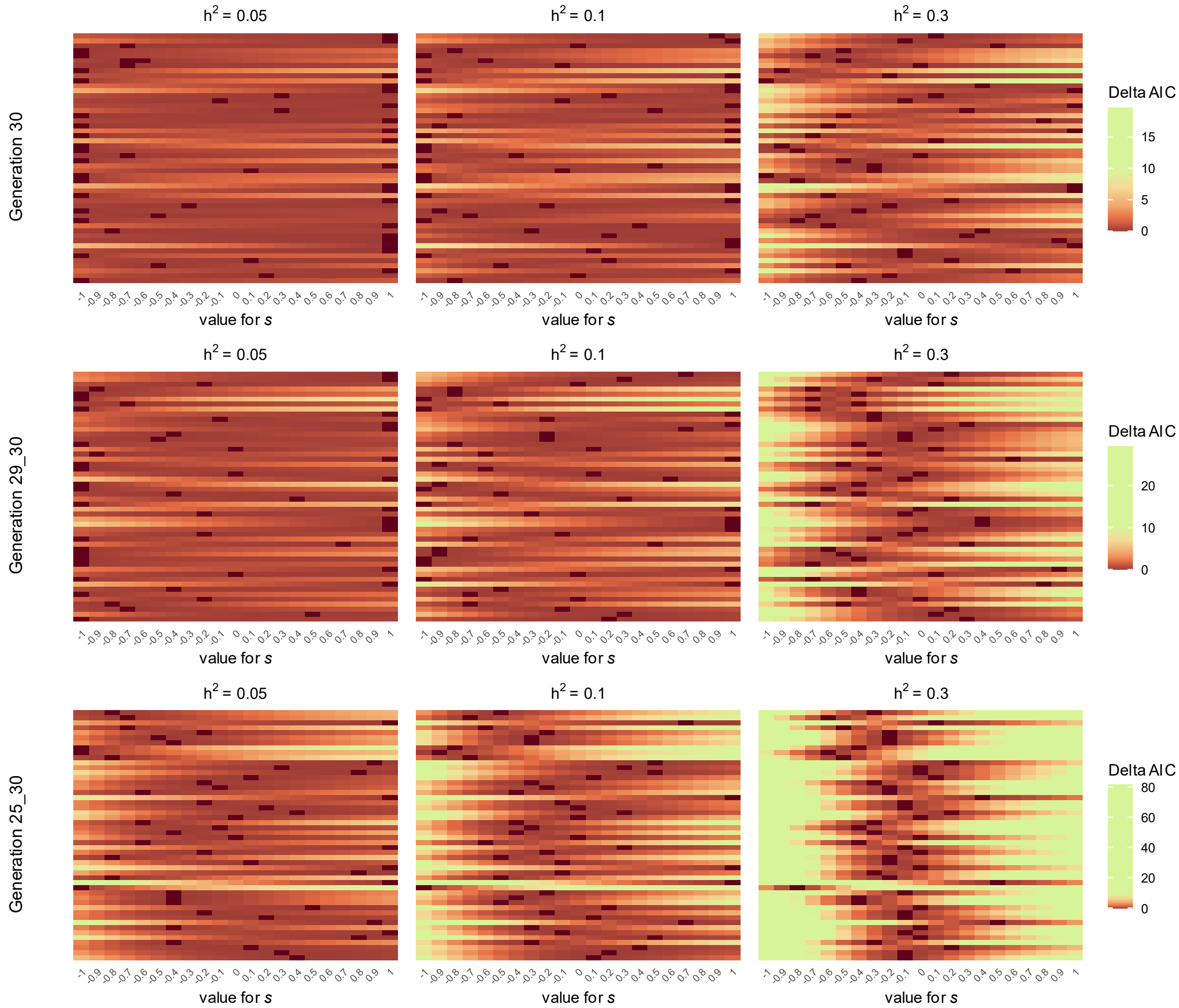


Supplementary Figure 2. $\Delta AIC_s$ scores for $s$-value estimation on random selection on a trait with a heritability of 0.05, 0.1 and 0.3. The results are given for generation 30, 29-30, and 25-30, the rows represent the 50 replicates for each selection scenario.

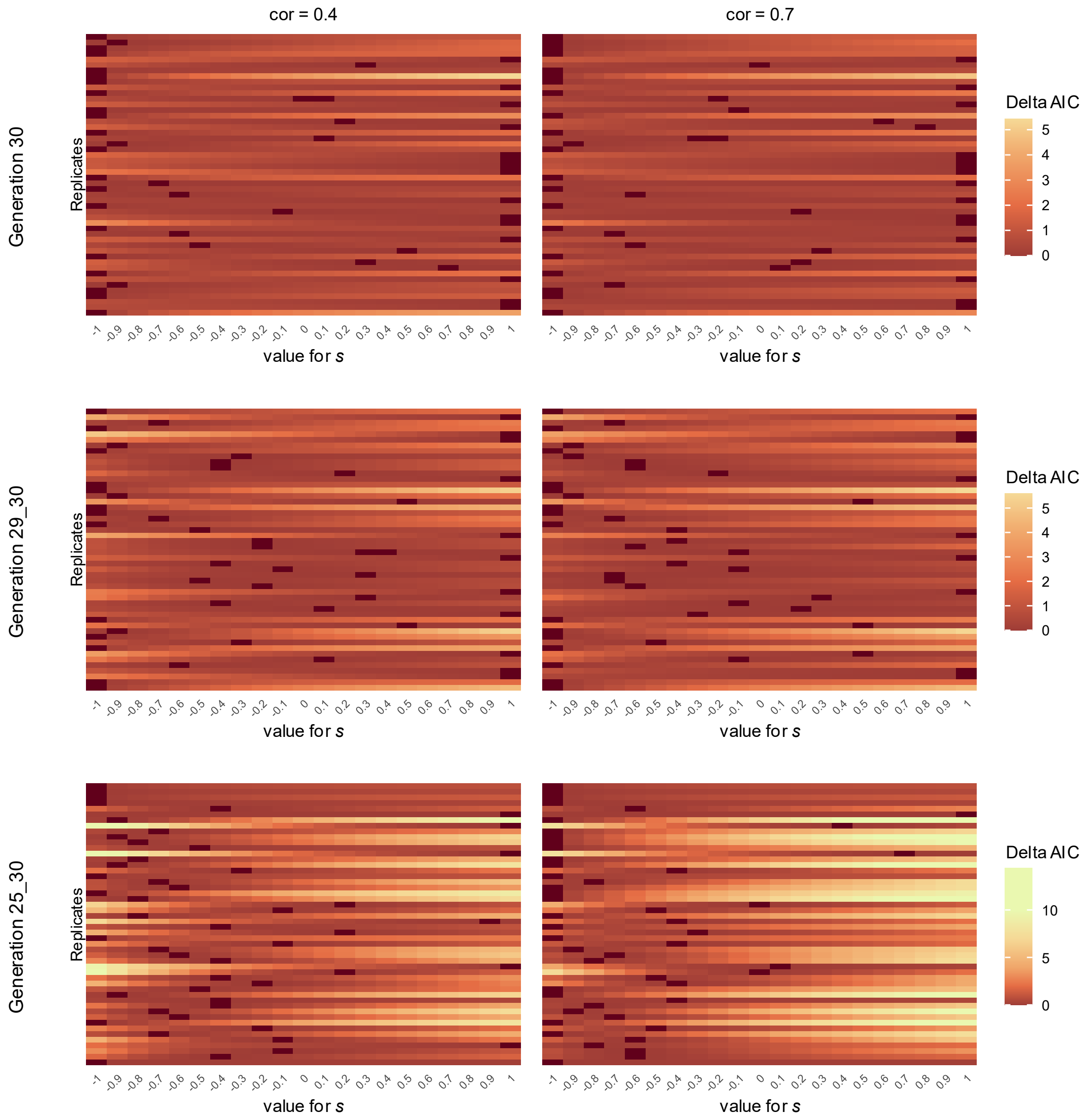


Supplementary Figure 3. $\Delta AIC_s$ scores for $s$-value estimation for indirect selection on a trait with a heritability of 0.05, by direct selection on a trait with a heritability of 0.1, with a correlation of 0.4 and 0.7. The results are given for generation 30, 29-30, and 25-30, the rows represent the 50 replicates for each selection scenario.

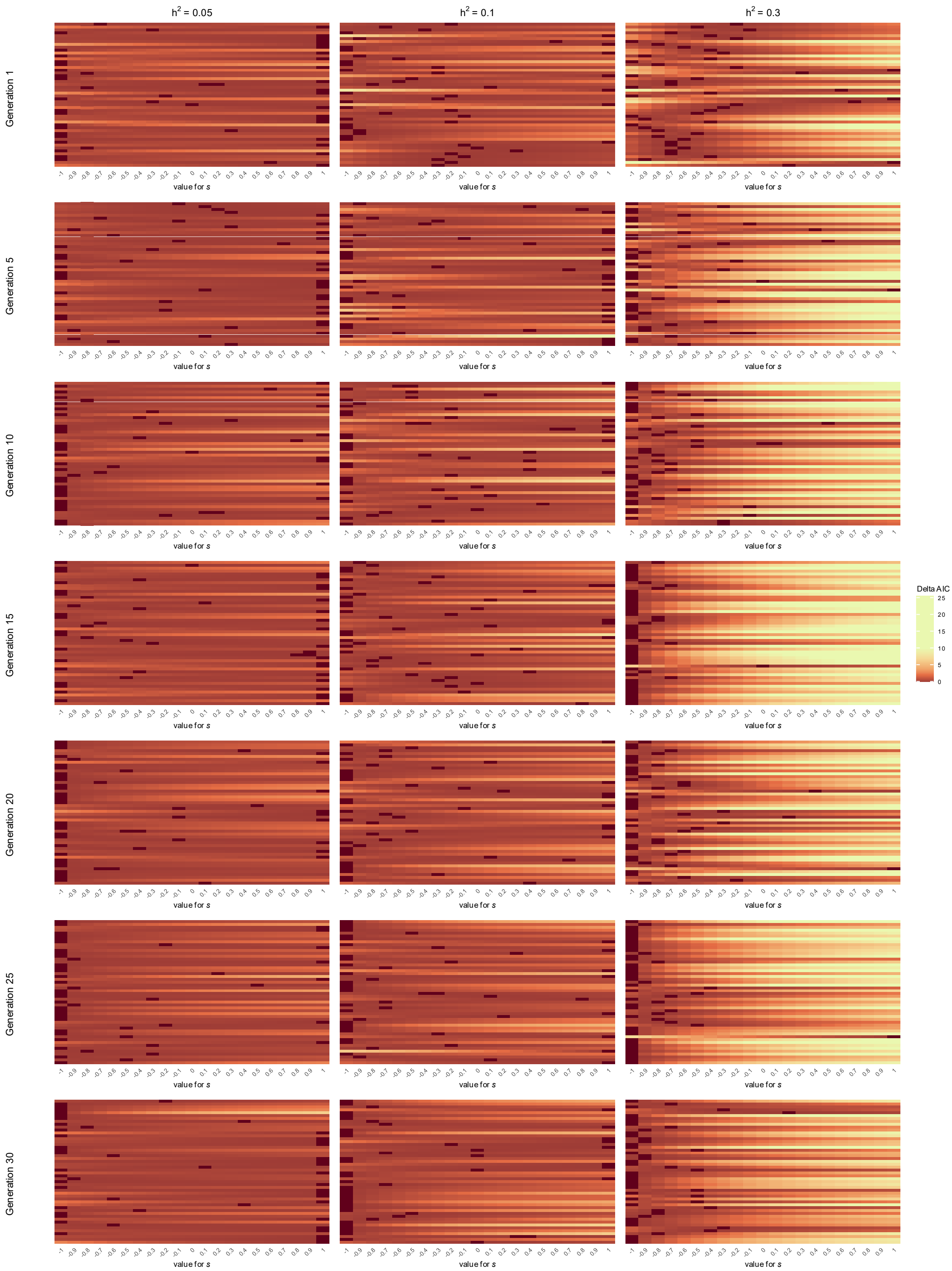


Supplementary Figure 4. $\Delta AIC_s$ scores for $s$-value estimation on directional selection on a trait with a heritability of 0.05, 0.1 and 0.3. The results are given for generation 1, 5, 10, 15, 20, 25, and 30. The rows represent the 50 replicates for each selection scenario.

Supplementary Table 1. Regression coefficients between MAF and causal loci for directional selection, with heritability 0.05.

| Generation | Mean slope (SD) | Mean adjusted $R^2$ (SD) | Mean *p*-value |
|---|---|---|---|
| Generation 1 | 0.031 (0.109) | 0.000 (0.001) | 0.473 |
| Generation 5 | 0.000 (0.107) | 0.000 (0.001) | 0.533 |
| Generation 10 | -0.045 (0.100) | 0.000 (0.001) | 0.491 |
| Generation 15 | -0.103 (0.103) | 0.001 (0.001) | 0.411 |
| Generation 20 | -0.181 (0.094) | 0.002 (0.002) | 0.187 |
| Generation 25 | -0.239 (0.096) | 0.003 (0.003) | 0.084 |
| Generation 30 | -0.297 (0.090) | 0.005 (0.003) | 0.025* |

Supplementary Table 2. Regression coefficients between MAF and causal loci for directional selection, with heritability 0.1.

| Generation | Mean slope (SD) | Mean adjusted $R^2$ (SD) | Mean *p*-value |
|---|---|---|---|
| Generation 1 | 0.047 (0.087) | 0.000 (0.001) | 0.538 |
| Generation 5 | 0.026 (0.100) | 0.000 (0.001) | 0.552 |
| Generation 10 | -0.055 (0.109) | 0.000 (0.001) | 0.434 |
| Generation 15 | -0.149 (0.109) | 0.001 (0.002) | 0.228 |
| Generation 20 | -0.251 (0.108) | 0.004 (0.003) | 0.077 |
| Generation 25 | -0.348 (0.102) | 0.007 (0.004) | 0.024* |
| Generation 30 | -0.430 (0.099) | 0.012 (0.005) | 0.003* |

Supplementary Table 3. Regression coefficients between MAF and causal loci for directional selection, with heritability 0.3.

| Generation | Mean slope (SD) | Mean adjusted $R^2$ (SD) | Mean *p*-value |
|---|---|---|---|
| Generation 1 | 0.064 (0.105) | 0.000 (0.001) | 0.393 |
| Generation 5 | 0.062 (0.093) | 0.000 (0.001) | 0.446 |
| Generation 10 | -0.027 (0.083) | 0.000 (0.001) | 0.501 |
| Generation 15 | -0.162 (0.085) | 0.002 (0.002) | 0.182 |
| Generation 20 | -0.300 (0.088) | 0.006 (0.003) | 0.025* |
| Generation 25 | -0.412 (0.086) | 0.013 (0.005) | 0.002* |
| Generation 30 | -0.512 (0.083) | 0.020 (0.006) | 0.000* |

Supplementary Table 4. Regression coefficients between MAF and causal loci for random selection, with heritability 0.05, 0.1 and 0.3.

| Generation | Mean slope (SD) | Mean adjusted $R^2$ (SD) | Mean *p*-value |
|---|---|---|---|
| Generation 1 | 0.015 (0.095) | 0.000 (0.001) | 0.582 |
| Generation 5 | 0.026 (0.105) | 0.000 (0.001) | 0.549 |
| Generation 10 | 0.028 (0.099) | 0.000 (0.001) | 0.540 |
| Generation 15 | 0.021 (0.107) | 0.000 (0.001) | 0.542 |
| Generation 20 | 0.016 (0.101) | 0.000 (0.001) | 0.556 |
| Generation 25 | 0.017 (0.098) | 0.000 (0.001) | 0.542 |
| Generation 30 | 0.024 (0.099) | 0.000 (0.001) | 0.542 |

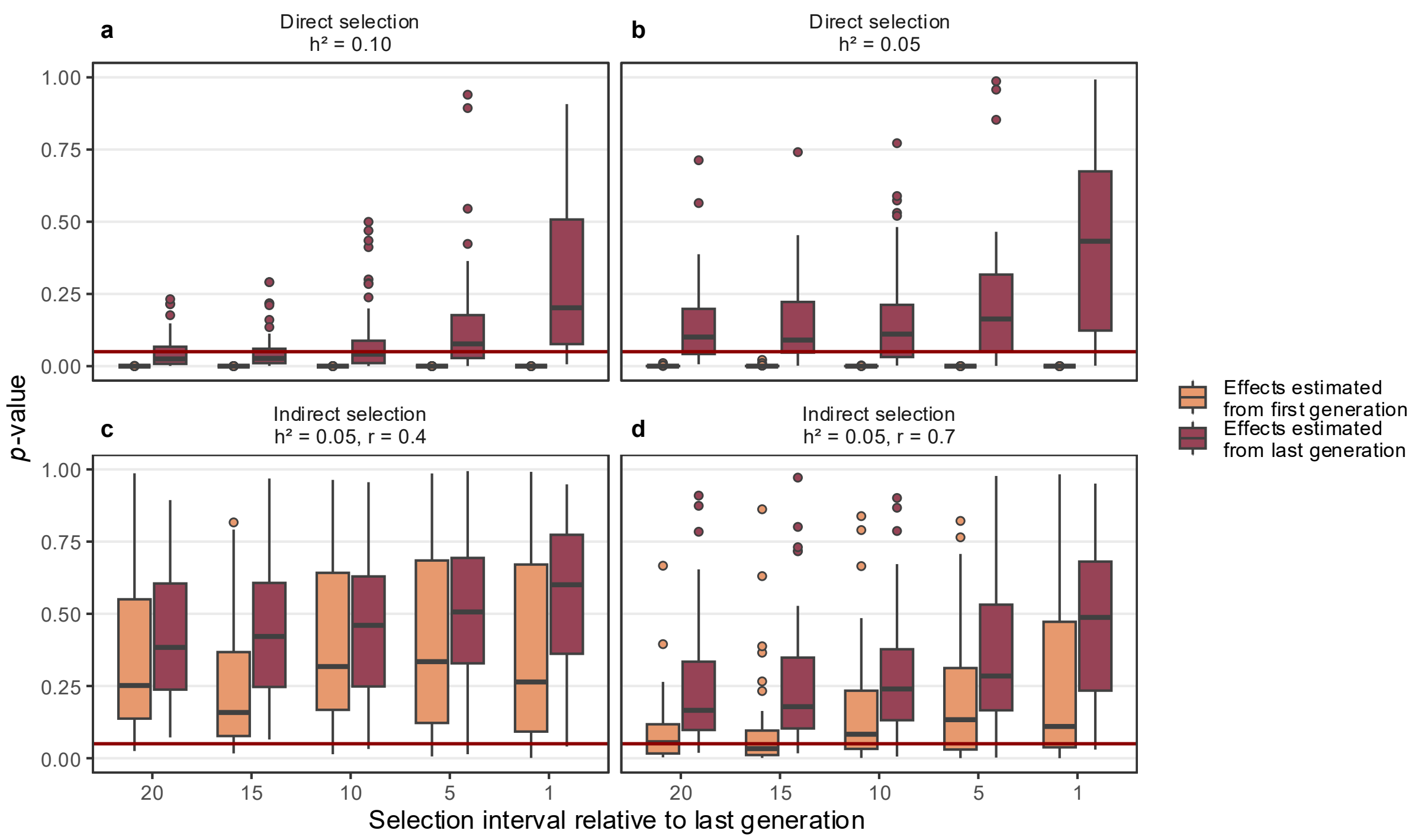


Supplementary Figure 5. Results for $\widehat{G}$ on direct selection with heritabilities 0.05 and 0.1 (a, b) and indirect selection with a correlation of 0.4 and 0.7 between the trait under direct selection ($h^2$=0.1) and the trait under indirect selection ($h^2$=0.05) (c, d). The boxplots show the *p*-values for 50 replicates for different selection intervals relative to the last generation (20, 15, 10, 5, 1). The red lines depict the significance threshold of $p = 0.05$.

Supplementary Table 5. Overview of number of replicates (out of 50) with a *p*-value < 0.05 for $\widehat{G}$ for directional selection with heritabilities 0.1 and 0.05, and indirect selection on a trait with heritability 0.05, which was correlated to a directly selected trait with heritability 0.1 with a correlation of 0.4 or 0.7. Results are given for both the estimated additive marker effects from the starting generation (Effect 1) or the last generation of the selection interval (Effect 2).

| | **Directional selection ($h^2$=0.1)** | | **Directional selection ($h^2$=0.05)** | | **Indirect selection ($h^2$=0.05, cor =0.4)** | | **Indirect selection ($h^2$=0.05, cor =0.7)** | |
|---|---|---|---|---|---|---|---|---|
| Selection interval | Effect 1[1] | Effect 2[2] | Effect 1 | Effect 2 | Effect 1 | Effect 2 | Effect 1 | Effect 2 |
| gen. 10-30 | 50 | 34 | 50 | 16 | 7 | 0 | 25 | 4 |
| gen. 15-30 | 50 | 35 | 50 | 14 | 8 | 0 | 30 | 3 |
| gen. 20-30 | 50 | 28 | 50 | 15 | 4 | 1 | 16 | 2 |
| gen. 25-30 | 50 | 19 | 50 | 13 | 6 | 1 | 17 | 2 |
| gen. 29-30 | 50 | 10 | 50 | 5 | 6 | 2 | 15 | 3 |

[1] Effect estimated in the first generations of the selection interval (10, 15, 20, 25, or 29); [2] Effect estimated in the last generations of the generation interval (30).
